# European Roma groups show complex West Eurasian admixture footprints and a common South Asian genetic origin

**DOI:** 10.1371/journal.pgen.1008417

**Published:** 2019-09-23

**Authors:** Neus Font-Porterias, Lara R. Arauna, Alaitz Poveda, Erica Bianco, Esther Rebato, Maria Joao Prata, Francesc Calafell, David Comas

**Affiliations:** 1 Institute of Evolutionary Biology (UPF-CSIC), Department of Experimental and Health Sciences, Universitat Pompeu Fabra, Barcelona, Spain; 2 Unit of Human Evolutionary Genetics, Institut Pasteur, Paris, France; 3 Department of Clinical Sciences, Genetic and Molecular Epidemiology Unit, Lund University Diabetes Centre, Lund University, Malmö, Sweden; 4 Department of Genetics, Physical Anthropology and Animal Physiology, University of the Basque Country UPV/EHU, Leioa, Spain; 5 Instituto de Investigacão e Inovacão em Saude/Institute of Molecular Pathology and Immunology of the University of Porto, Porto, Portugal; Faculty of Sciences, University of Porto, Porto, Portugal; CNRS - MNHN, FRANCE

## Abstract

The Roma population is the largest transnational ethnic minority in Europe, characterized by a linguistic, cultural and historical heterogeneity. Comparative linguistics and genetic studies have placed the origin of European Roma in the Northwest of India. After their migration across Persia, they entered into the Balkan Peninsula, from where they spread into Europe, arriving in the Iberian Peninsula in the 15th century. Their particular demographic history has genetic implications linked to rare and common diseases. However, the South Asian source of the proto-Roma remains still untargeted and the West Eurasian Roma component has not been yet deeply characterized. Here, in order to describe both the South Asian and West Eurasian ancestries, we analyze previously published genome-wide data of 152 European Roma and 34 new Iberian Roma samples at a fine-scale and haplotype-based level, with special focus on the Iberian Roma genetic substructure. Our results suggest that the putative origin of the proto-Roma involves a Punjabi group with low levels of West Eurasian ancestry. In addition, we have identified a complex West Eurasian component (around 65%) in the Roma, as a result of the admixture events occurred with non-proto-Roma populations between 1270–1580. Particularly, we have detected the Balkan genetic footprint in all European Roma, and the Baltic and Iberian components in the Northern and Western Roma groups, respectively. Finally, our results show genetic substructure within the Iberian Roma, with different levels of West Eurasian admixture, as a result of the complex historical events occurred in the Peninsula.

## Author summary

Human demographic processes and admixture events leave traceable footprints in the genomes of the populations and they can modulate the genetic architecture of complex diseases. Here, we aim to study the Roma people, an admixed population with a particular demographic history recognized as the largest ethnic minority in Europe. Previous studies suggest that they originated in South Asia 1,500 years ago and followed a diaspora towards Europe with extensive admixture with non-Roma West Eurasian groups. However, the genetic components of the Roma have not been deeply characterized. Our study reveals a common South Asian origin of all European Roma, closely related to a Punjabi group from Northwestern India. Through fine-scale haplotype-based methods, we describe a complex West Eurasian genetic component in the Roma groups, identifying a common Balkan ancestry and country-specific admixture footprints consistent with the dispersion through Europe. Our findings provide new insights into the demographic history and recent admixture events that have shaped the genetic composition of European Roma groups and could enable a better genetic characterization of complex disease in this population.

## Introduction

The diaspora of the Roma people, also known with the misnomer of Gypsies, is a not-well documented human movement, which is characterized by recent dispersals and multiple founder events. The Roma population is recognized as the largest transnational ethnic minority in Europe, with an estimated population of up to 10 million, although their exact number is difficult to estimate due to the lack of reliable census surveys. They consist of a heterogeneous and substructured mosaic of populations that differ linguistically, culturally, historically, and in their relation to nearby populations [1]. Their demographic history together with their endogamous social practices [1] have contributed to a particularly different spectrum of Mendelian disorders when compared with other neighboring European populations [2,3]. Historical records confirm the persecution and social marginalization that this population has suffered since their arrival to Europe [1].

Comparative linguistics has placed the origin of European Roma in India, particularly in the northwestern region, as Romani is closely related to Punjabi and Kashmiri languages [4,5]. However, the social organization and cultural dynamics in Indian populations lead to substructure in closely-related linguistic groups, as is reflected in the different proportions of Ancestral North Indian (ANI) and Ancestral South Indian (ASI) genetic components [6] shown in groups even living in the same geographic region, which prevents them to be considered as genetically homogeneous groups [7] and challenges the retrieval of the origins of Roma based solely on linguistic data. The Indian genetic component of the Roma population was first proposed after the identification of shared disease-causing mutations with Indian and Pakistani patients [8,9]. In addition, analyses of uniparental markers permitted to assign an Indian origin for some maternal and paternal lineages found in the Roma [10–12], namely those belonging to the M-haplogroups (M5, M18, M25, M35) in the mitochondrial DNA [13], and to H-M69 in the Y-chromosome [14]. Furthermore, genome-wide studies indicate that the European Roma originate from a reduced number of founders (proto-Roma) whose ancestral homeland was the current Punjab state of India [10,15,16].

According to previous historical and anthropological evidence, a subsequent migration from Northwest India through Persia and Armenia preceded the entrance in the Balkans, from where they spread across the entire Europe. During the 11th and 12th centuries, some Roma settled in the surroundings of the Ottoman Empire, in the Balkan Peninsula (Balkan Roma), other groups spread across the Danubian Principalities (present-day Romania, Moldova, and Hungary), where they were forced into slavery (Vlax Roma), while the Romungro group started a dispersion across the Austro-Hungarian Empire [2]. Finally, other small groups moved into North, Central, and Western Europe (Northwestern Roma), having arrived into Iberia in the early 15th century, as document a number of Iberian historical records mentioning the presence of Roma groups in Zaragoza and Barcelona in 1425 and 1447, respectively [17]. The Roma diaspora through the Middle East, Caucasus, and Europe was a very complex process during which the emerging pattern of genetic substructure was highly influenced by differential gene flow from different West Eurasian (European, Middle Eastern and Caucasian) non-Roma populations [15,18] and even within Roma groups [19]. Genome-wide data showed that the Roma genomes harbor around 80% of Western Eurasian ancestry, while the remaining ancestry is from South Asian sources [16]. However, this estimate of the West Eurasian component is not only derived from their recent (post-exodus) admixture with non-Roma Europeans, as prior to their arrival into Europe, Roma might already carried an Ancestral West Eurasian (AWE) component from South Asian sources [16], due to admixture events that occurred in South Asia around 1,900–4,200 years ago (ANI component) [20], thus before the proto-Roma people left South Asia.

However, previous genetic studies of the European Roma, despite the wealth of insights provided into their demographic history, show multiple limitations. First, South Asian populations have been primarily studied using the linguistic affiliation as criteria to classify individuals into groups, which often conflicts with genetic intra-group homogeneity. Second, the European Roma population has been approximated as a simple admixture between South Asian and European sources, without a detailed analysis of the West Eurasian component in Roma. In addition, most of the analyses relied in allele frequency-based methods, yet haplotype-based approaches provide a fine-scale characterization, and perform better than allele frequency analyses in populations that have been under strong genetic drift [21,22]. Finally, there are still few studies focused on the Iberian Roma population, which represents the westernmost expansion of the Roma diaspora in Eurasia. To overcome the mentioned limitations, the present study consists of a genome-wide analysis of the European Roma (including new samples from the Iberian Roma), with the following aims: (i) to shed light on the South Asian origin of the proto-Roma population; (ii) to assess the level of admixture of the Roma with other European populations as well as with Middle Easterners and North Africans; and, (iii) to characterize the patterns of genetic substructure among the Iberian Roma. Our analysis unravels at fine-scale the genetic components of European Roma groups, dissecting the original South Asian, ancestral West Eurasian, and recent European components.

## Results

### European Roma genetic substructure

The European Roma population was first assessed in a worldwide context (Dataset1, S1 Table, see Materials and Methods). A Principal Component Analysis (PCA) was performed with samples from Europe, Africa, Middle East, Caucasus, Central and South Asia. Roma samples fall between non-Roma European and South Asian populations (S1 Fig), in agreement with their demographic historical records [1] and previous genetic studies [15]. In addition, ADMIXTURE results further confirm PCA results, as at k = 3, European Roma show a combination of two cluster components (dark red and dark blue) mainly found in South Asian and West Eurasian samples. At k = 6 (lowest cross-validation error value), the Roma individuals displayed membership in a specific cluster and a yellow component mainly found in southwestern Eurasia, which reproduces previous results [15] (S2 Fig).

**Fig 1:**
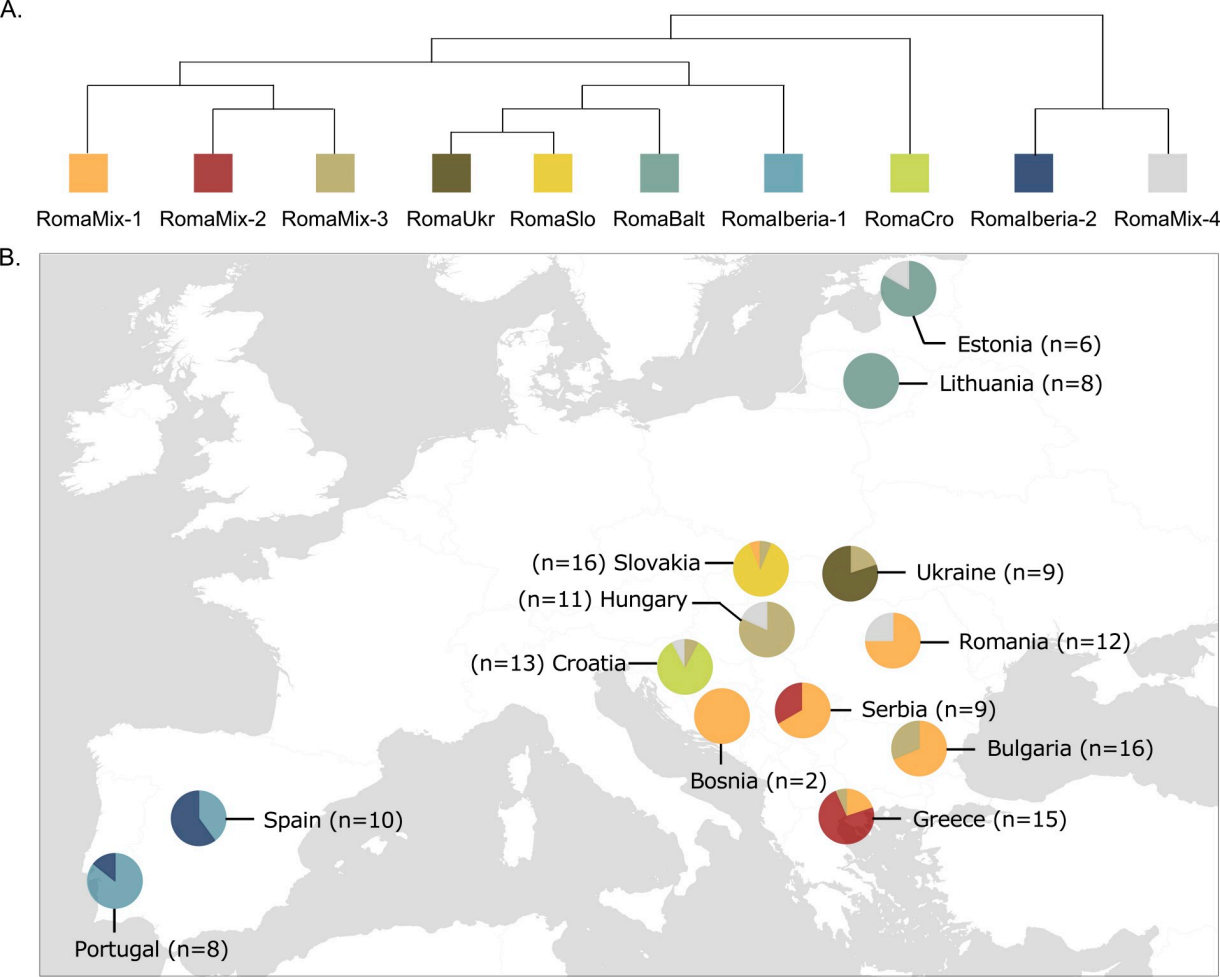
European Roma substructure (Dataset1). (A) European Roma fineSTRUCTURE dendrogam showing the 10 European Roma clusters. (B) European Roma sample location and sample size (pie charts are colored according to the clusters in A).

To further describe the Roma genetic substructure and to reveal fine-scale patterns, we used haplotype-based methods: ChromoPainter and fineSTRUCTURE. Most European Roma samples cluster together in a sister clade of MiddleEast-Caucasus and Europe super-group (S3 Fig). These Roma samples belong to ten different clusters correlated with geography, grouping together individuals from the same European regions (North, West, Central, and Balkans) (Fig 1A and 1B). As shown in the dendrogram (Fig 1A) and based on the Total Variance Distance (TVD) values, the most significantly differentiated Roma clusters are RomaIberia-2 and RomaMix-4 (p < 0.001) (S4 Fig, S2 Table). The non-Roma reference samples were classified in 51 genetic clusters from four different large super-groups (Europe, MiddleEast-Caucasus, Central-SouthAsia, and MiddleEast-Africa) (S3 Fig).

### Admixture in Roma and South Asian origin of the proto-Roma population

**Fig 2:**
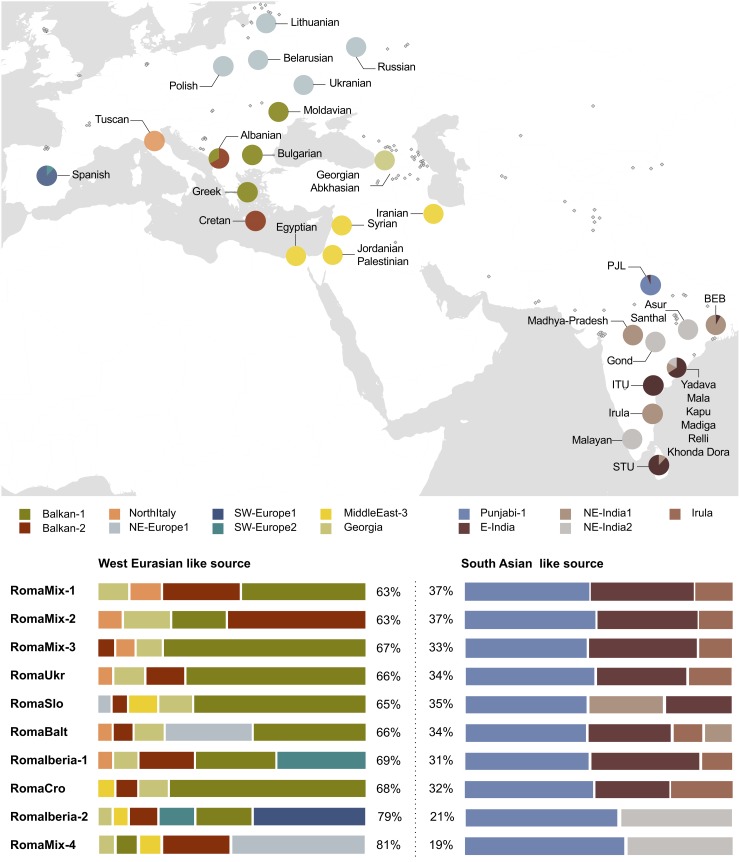
West Eurasian and South Asian ancestry of the European Roma (Dataset1) from GLOBETROTTER results. Pie charts on the map show the geographic location of the donor populations. Grey diamonds display those samples that do not contribute to the Roma ancestry. For each Roma cluster, the major source (West Eurasian like) and minor source (South Asian like) are shown: the proportion (in percentage) of each source and a horizontal bar with the proportions of the donor populations in each source. Only donor groups that contribute a minimum of 3% to the Roma genomes are shown. 1000G population labels are used in the map for ITU (Indian Telugu from the UK), STU (Sri Lankan Tamil from the UK), BEB (Bengali from Bangladesh), PJL (Punjabi from Lahore, Pakistan).

Admixture events that have shaped the genetic composition of the Roma population were inferred with GLOBETROTTER. For all European Roma clusters, “one-date” type of admixture event (single admixture date between two sources) was detected involving two sources: a West Eurasian-like major source and South Asian-like minor source, around 1270–1580 (S3 Table, Fig 2, Table 1). This interval of admixture dates overlaps with the period when the first historical records report the presence of Roma groups in each European country, although these records represent the lower limits for the actual first Roma settlements. In general, Roma from the surroundings of the Balkan Peninsula and Central Europe (RomaMix-1, RomaMix-2, RomaMix-3, RomaUkr) have earlier admixture dates (Table 1), which supports the dispersion into Europe via the Balkans [15].

**Table 1:** Admixture dates inferred by GLOBETROTTER for each European Roma cluster with each estimated date and 95%CI from 100 bootstrap resamples in generations ago (GA) and years CE (considering a generation time of 25 years). The first historical records of Roma presence in each sampled European country [1,60,61] are shown with the same assumptions as in [15].

Recipient cluster	Date (GA)	CI 95% (GA)	Date (CE)	CI 95% (CE)
RomaMix-1	27.286	19.761–31.705	1318	1207–1505
RomaMix-2	25.103	20.664–33.405	1372	1164–1483
RomaMix-3	23.001	17.589–31.596	1425	1210–1560
RomaUkr	24.867	21.134–30.97	1378	1225–1471
RomaSlo	18.465	15.089–24.039	1538	1399–1622
RomaBalt	20.752	16.726–24.971	1481	1375–1581
RomaIberia-1	24.269	16.883–30.228	1393	1244–1577
RomaCro	19.969	14.959–25.845	1501	1353–1626
RomaIberia-2	17.905	12.668–28.058	1552	1298–1683
RomaMix-4	18.589	9.858–30.801	1535	1229–1753

1^st^ historical records of Roma presence: Serbia (1348), Croatia (1362), Bulgaria (1378), Romania (1385), Greece (1386), Hungary (1402), Spain (1425), Slovakia (1444), Portugal (1462), Ukraine (1501), Lithuania (1501), Estonia (1540)

Regarding the South Asian-like source, it contributes around 35% to the admixture and its most representative cluster is Punjabi-1, from Northwestern India, (Fig 2, S3 Table). Although Punjabis have a linguistically uniform identity [23], they are genetically heterogeneous. In fact, Punjabi samples do not cluster together, instead they are spread along PC2 (S1 Fig), as well as in the fineSTRUCTURE dendrogram (S3 Fig), with three different Punjabi clusters with increasing levels of ANI component (S5 Fig, S4 and S5A Tables). Thus, most of the South Asian ancestry of the Roma is mainly shared with the group of individuals from Punjab with less West Eurasian component (Punjabi-1, S3 Table).

The rest of South Asian surrogates identified in the minor source correspond to southeastern Dravidian-speaking populations (E-India, Irula clusters) (Fig 2, S3 Table), which also exhibit low levels of West Eurasian ancestry (S5 Fig, S5A Table).

Altogether, these findings suggest that the most likely proxy for the South Asian origin of the proto-Roma, is the ancestral source here described as a mixture of present-day South Asian groups with a low West Eurasian signature.

### Recent West Eurasian admixture

The West Eurasian-like source contributes around 65% to the admixture event. This component captures the recent West Eurasian admixture between the proto-Roma and West Eurasians during their diaspora from India to Europe, in other words, it does not include the AWE component present in South Asian populations (S1 Note, S6 Fig) estimated to be around 15% (S5B Table). This recent West Eurasian ancestry is lower in the Roma groups from the Balkan Peninsula and Central Europe (RomaMix-1 and RomaMix-2), around 60%, and it increases up to 80% (RomaIberia-2) as the distance from the Balkans increases (Fig 2, S3 Table).

The main contribution of this major source is from southeastern European clusters (Balkan-1 and Balkan-2), with this area being the historically reported gateway of the Roma groups into Europe [1]. The component from Middle East and Caucasian clusters was found to be moderate in the Roma groups. Besides these two components, additional distinct European ancestries are detected in the Northwestern Roma groups from the Baltic (Estonia-Lithuania) and Iberia (Spain-Portugal). Specifically, while RomaBalt cluster shows a northeastern European component (NE-Europe1 cluster), RomaIberia-1 and RomaIberia-2 contain a southwestern European component (SW-Europe1 and SW-Europe2) each. This result indicates that, in the Roma groups that migrated to Northern and Southwestern Europe, the Balkan component left a footprint still clearly detectable today, though having been highly reconfigured by admixture in the Baltic region and the Iberian Peninsula, respectively (Fig 2, S3 Table).

Regarding the Iberian Roma, the samples constitute two highly differentiated clusters (RomaIberia-1 and RomaIberia-2) not found elsewhere, which suggests a deep genetic substructure within the Roma settled in Iberia (Figs 1 and 2, S3 Table).

### Sex-biased gene flow

As mentioned above, the European Roma ancestry contains two main sources: the West Eurasian (European and MiddleEast-Caucasus) and the South Asian components. However, these ancestry proportions differ significantly when comparing the X chromosome to the autosomes: the South Asian ancestry is significantly higher in the X chromosome while the MiddleEast-Caucasus proportion is significantly higher in the autosomes (S6 Table, S7 Fig). These results point to a sex-biased admixture during the Roma diaspora, likely characterized by a higher influx of non-Roma males than females from the Middle East and Caucasus. The proportions of European ancestry contained in the autosomes and the X chromosome are similar, although RomaBalt, RomaIberia-1, RomaIberia-2 and RomaMix-4 show higher levels of European ancestry in the autosomes. These findings can also indicate different sex-biased gene flow processes in the European Roma groups, which might be the result of different social patterns among groups. Future studies with mtDNA and Y- chromosome data could add further insights into these results, as well as sex-specific fertility inheritance processes in the Roma population [24].

### Roma demographic patterns

To investigate the effective population size (Ne) dynamics, we have estimated the Ne of each Roma group and the ancestry-specific Ne. On one hand, all Roma groups show a long uninterrupted Ne decrease followed by an increase of Ne (without reaching the levels of the NorthItaly cluster, which we used as a European reference) (S8 Fig). The change of the Ne trend is slightly correlated with the start of the admixture in each Roma group (S9 Fig), which might point to the gradual settlement of the Roma population in Europe. On the other hand, we inferred Ne through time for the three ancestral Roma source populations (European, MiddleEast-Caucasus and SouthAsian), focusing on their Ne before the admixture: 34 generations ago, as the more ancient lowest confidence interval (CI) inferred from GLOBETROTTER is found in RomaMix-2 at 1164 CE (S7A Table). The European Ne_g = 34_ is 2.12 to 2.64 times higher than the South Asian Ne_g = 34_, which is 1.27–1.43 times higher than the MiddleEast-Caucasus Ne_g = 34_ (S7B Table). In contrast, the fold-change between the European and South Asian ancestry proportions is lower than 2 in all Roma groups (except RomaIberia-2 and RomaMix-4) and between South Asian and MiddleEast-Caucasus ancestry proportions is higher than 1.5 fold in all Roma groups (S7C Table). These differences between the ancestry proportions and the ancestry-specific Ne could be explained by the fact that a small South Asian proto-Roma group of founders had a continuous gene flow with different non-related groups from the MiddleEast and Caucasus and different non-Roma European populations, during their West Eurasian diaspora (see S4 Note).

Runs of homozygosity (ROH) were computed to assess the levels of inbreeding and the degree of genetic isolation in the Roma groups. In general, the mean ROH length of the Roma groups is significantly higher than the mean of the non-Roma reference Balkan-2 and Punjabi-1 clusters. For all ROH length categories, Roma groups present similar values than those of Kalash (S10 Fig, S8A Table), which is known to be a highly inbred population [25], possibly due to genetic isolation, although their isolation degree is in debate [26,27]. The average ROH lengths of the Roma maintain high values after a first significantly decrease between the first and the second categories (1–2 and 2–3 Mb, respectively) (S8B Table), which suggest that the inbreeding signals of Roma are the result of a continuous, although decreasing, level of isolation, from historical to recent times. Furthermore, the Roma groups with more West Eurasian ancestry (IberianRoma-2 and RomaMix-4) are the clusters with the lowest mean ROH length values across all categories (S10 Fig). Thus, these results additionally evidence a degree of heterogeneity within Roma from the Iberian Peninsula that need to be further investigated.

### Iberian Roma genetic characterization

#### Iberian Roma substructure

To further explore the genetic structure of the Iberian Roma population, we included 34 newly genotyped Roma samples from the Iberian Peninsula (Dataset2, S9 Table, see Material and Methods). These samples fall between European and South Asian populations in the PCA (S11 Fig), the ADMIXTURE analysis (S12 Fig), and the fineSTRUCTURE dendrogram (S13 Fig), in agreement with the above results using European Roma individuals (Dataset1). Iberian Roma samples were classified in four different genetic clusters (S14 Fig). Although the four Iberian Roma groups are only partially clustered by geography, different patterns are discerned: IberianRoma-1 and IberiaRoma-2 contain samples from the northeastern region of the Iberian Peninsula, IberianRoma-3 is restricted to the south, and IberianRoma-4 is mainly formed by samples from the northwestern region (S14A and S14B Fig). As shown in the dendrogram (S14B Fig), IberianRoma-4 is the most significantly differentiated group (p < 0.001) (S15 Fig, S10 Table).

Analogously to Dataset1 dendrogram (S3 Fig), the non-Roma reference samples were classified in 83 clusters, which can be summarized in four large super-groups (MiddleEast-Africa, Europe, MiddleEast-Caucasus, and Central-SouthAsia) (S13 Fig).

#### Recent West Eurasian admixture in Iberian Roma groups

Admixture events in the Iberian Roma clusters were inferred with GLOBETROTTER. As shown above for the general Roma groups (Dataset1), one admixture event between a West Eurasian-like major source and a South Asian-like minor source was detected in each of the four Iberian Roma groups (S11 Table). The date intervals (95% CI) of the inferred admixture event for each Iberian Roma cluster are: 1210–1557 (IberianRoma-1), 1241–1536 (IberianRoma-2), 1279–1583 (IberianRoma-3), and 1532–1730 (IberianRoma-4), having the latter the most recent dates (S16 Fig).

Regarding the minor source, the most contributing clusters are Punjabi-1, E-India, NE-India and Irula (S11 Table), as observed in Dataset1, which fits the hypothesis that the Roma origin can be placed in a group of South Asian individuals with low West Eurasian ancestry.

**Fig 3:**
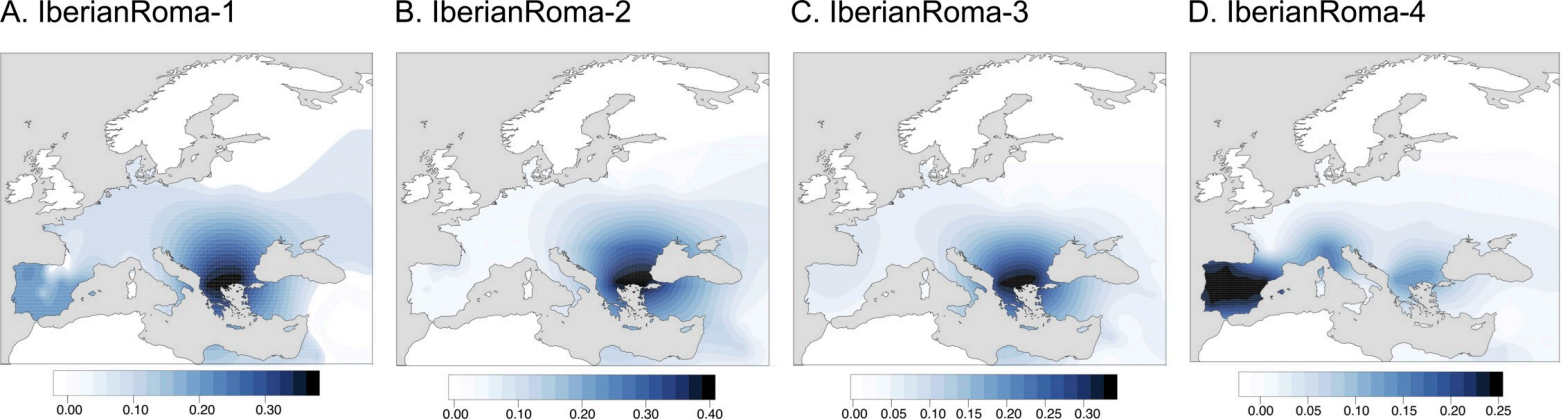
West Eurasian ancestry of the Iberian Roma (Dataset2). Kriging model of the spatial distribution of the major source donor proportion inferred with GLOBETROTTER, reflecting the West Eurasian ancestry proportions in each Roma group (A-D).

The West Eurasian-like source mainly consists of Balkan and Southwestern European clusters (SW-Europe2, SW-Europe3, and Basque) and, in less proportion, Middle Eastern and Caucasian populations (Egypt-Bedouin, W-Caucasus2, and Georgia) (Fig 3, S14C Fig, S11 Table), which reinforces the evidence of the three main focus of migration of the Iberian Roma: their way out from Northwestern India, the entrance into Europe from the Balkan Peninsula, and the arrival into the Iberian Peninsula. Although the surrogate populations involved in the admixture event of the four Iberian Roma groups are similar, some distinctness can be appreciated. IberianRoma-4, as mentioned above, is the most differentiated group and GLOBETROTTER results suggest that it is due to the different source and proportion of European ancestry: first, the contribution of Southwestern European clusters is higher than in the rest of the Iberian Roma clusters; and second, other European clusters (NorthItaly, E-Europe2, and NW-Europe2) are also identified, but they are absent in the rest of Iberian Roma groups (Fig 3, S14C Fig, S11 Table). The inferred IberianRoma-4 admixture event is the only one that contains Balkan and Middle East surrogates in the minor source, possibly as a result of its high non-Roma European ancestry (S11 Table). Moreover, IberianRoma-3 exhibits some degree of Northwest African admixture (~1%), probably due to its southern location in the Iberian Peninsula (S14C Fig, S11 Table), where historically the North African gene flow into the general Iberian population was more relevant [28,29]. Besides, IberianRoma-3 is also the group with more NE-Europe2 (~2%) (S14C Fig, S11 Table). IberianRoma-2 contains exclusively Roma samples from the Basque country and, accordingly, it shows the highest non-Roma Basque ancestry. Altogether, these results confirm the presence of genetic substructure and differential admixture within the Iberian Roma population, revealing four distinct patterns of spatial distribution (Fig 3), and, furthermore, reject a putative North African origin of the Iberian Roma groups [30].

#### Demographic patterns in Iberian Roma

Overall, Iberian Roma show a significantly higher mean ROH length than the non-Roma reference European populations (Basque and SW-Europe2) and the Punjabi-1 cluster. At larger ROH length categories, Iberian Roma present higher values than Kalash (S17 Fig, S12A Table). In addition, some specific trends can be recognized in the Iberian Roma groups. Namely, the progressive decline of ROH length in IberianRoma-4 is significantly different from the rest of Roma groups and it mirrors the SW-Europe2 one, being their differences not significant (S12A Table). On the other hand, IberianRoma-2 exhibits a sudden decrease of ROH length at 4–5 ROH category, although differences are not significant probably due to their low sample size (S12B Table); while IberianRoma-1 and IberianRoma-3 show high levels of inbreeding (significant p-values only between the 1-2Mb category and the rest of ROH length categories), suggesting different degrees of relatedness in the Iberian Roma groups.

The Ne estimations through time in each Iberian Roma group are lower than the ones from SW-Europe2, and a constant Roma Ne reduction is detected from around 750 to 1600 (S18 Fig). This Ne reduction trend is reversed after the admixture event inferred by GLOBETROTTER. These results agree with the ones obtained for Dataset1, which contains all European Roma groups.

## Discussion

The demographic history of the Roma population is characterized by a series of bottlenecks and admixture events that have occurred since the proto-Roma left India, after their arrival to the Balkans and spread throughout Europe, and in the case of Iberian Roma, after their settlement in the Iberian Peninsula. The study of their genetic profile in a worldwide context places them between South Asians and Europeans, which confirms previous findings of admixture [10,15,16]. A fine-scale approach has allowed us to distinguish the recent West Eurasian component, which is the result of the admixture with non-Roma West Eurasian populations. Our estimates of this recent West Eurasian component, around 65%, are lower than the previously reported 80% [16], as it only includes the “post-exodus from India” admixture and not the “pre-exodus from India” AWE component (around 15% based on the f4 ratio estimates). This recent West Eurasian component was acquired between 1270–1580. Although GLOBETROTTER infers this admixture as a single pulse event (“one-date”), it would require large datasets to distinguish continuous from single pulse admixture [31].

Regarding the origin of the proto-Roma population, Northwestern India has been previously proposed as the putative source of their South Asian ancestry [4,5]. Although it is a geographically well-defined area, their populations are socially, linguistically, and genetically heterogeneous, with high levels of stratification and substructure: their lands comprise from tribe clans to upper-caste groups, and from Dravidian to Indo-European speaking groups [32]. Our analyses show that they are dispersed along the PC with different admixture proportions (S1–S3 and S5 Figs). Within the boundaries of Northwestern India, the Punjab region has been further placed as the ancestral homeland of the proto-Roma, through different approaches: identity by descend (IBD) sharing analyses [16], Approximate Bayesian Computation models [15], and mitochondrial M lineages [10] and tau haplotype [33] comparisons between Roma and South Asians. However, the linguistic identity that characterizes the Punjabi population is independent of their historical origin and social designation [23]. Punjab is a strategic region that has suffered repeated invasions from different sources [32], explaining why nowadays encompasses heterogeneous population with differential admixture and ancestral components. We have shown that the Punjabi samples are genetically heterogeneous, which mainly differ in the proportion of West Eurasian ancestry, further confirming previous results [7]. Our results add in the indication that the original genetic composition of the proto-Roma seems nearest to that of the Punjabi cluster from the less West Eurasian admixed group. Assuming that the individuals from this Punjabi cluster were already in Punjab when the rest of Punjabi clusters admixed with West Eurasians, socio-historical factors might have determined their differential admixture. In other words, this Punjabi cluster might derive from Punjabis who belonged to a lower caste group, since in agreement with previous studies, Indian lower caste groups are characterized by less West Eurasian admixture [6,7]. In addition, we have reported that Dravidian-speaking populations with high ASI ancestry (i.e. E-India and Irula clusters) are also involved in the South Asian source of the Roma individuals. These two sources of South Asian ancestry could solve the contradiction regarding the identification of uniparental Roma lineages with a Northwestern Indian origin [11] and the high Y-STR haplotype sharing among Roma and South Indian populations [34], as these findings could be explained by two overlapping scenarios. The first one, first mentioned by Turner [4], consists in considering a previous migration of nomadic groups into Northwestern India from Central India around 250 BCE and, after several centuries in Punjab with few external admixture, a single group of proto-Roma individuals left India. The second scenario refers to the fact that the genomes of present-day North Indians have more West Eurasian ancestry due to subsequent gene flow from West Eurasians after the proto-Roma left India [20], which explains the combination of populations with low West Eurasian ancestry identified in the South Asian Roma component. These two scenarios fit the idea that the Roma people descend from a single initial founder population [15].

After the exodus from India and during the diaspora through West Eurasia, the Roma population admixed with multiple non-Roma European, Middle Eastern and Caucasian groups. First, the European Roma ancestors arrived to Armenia through Persia [1]. Our results agree with a moderate Middle East and Caucasus gene flow during a rapid migration across this territory [15], specifically, we detect a higher rate of male gene flow, which could be related to the incorporation of Persian nomadic groups with the Roma [1]. Then, historical records suggest that, in Armenia, they followed the same route as the displaced Armenians towards Anatolia, due to the Mongol and Seljuq invasions (a Persian Muslim dynasty), from where they were pushed to the west until their entrance into Europe through the Thrace region in the Balkan Peninsula [35]. They settled in the Balkans for almost 200 years [35], where the Greek impact on the Romani language was much more extensive than the Persian [1]. Accordingly, we have identified the Balkan admixture footprint in the European Roma genomes with an ancestry gradient correlated with the distance to the Balkans: from 45% in Bulgarian, Greek, and Serbian Roma; to 25% in Lithuanian, Estonian, and Iberian Roma, which is further evidence that the dispersion into Europe took place via the Balkans [15]. After subsequent migrations and dispersions across Europe, Roma groups reached Northeastern Europe (e.g. Lithuania and Estonia) and Southwest Europe (e.g. Iberian Peninsula), at the beginning of the 16th and 15th centuries, respectively [1]. Particularly in these groups, we have identified the Baltic and Iberian components besides the common Balkan component.

In relation to the demographic dynamics, we have shown that the Ne reduction of the Roma groups ceased after the start of the admixture event, which points to the settlement of Roma in Europe and the beginning of more intense assimilation politics during the seventeenth century [1]. The Ne estimates (as discussed in S3A Note) might reflect Ne changes in the Roma groups due to a population expansion or the non-Roma West Eurasian admixture. In addition, the levels of inbreeding in the Roma population are higher than in non-Roma Europeans and similar to those of South Asian groups, which could be the result of endogamy practices and/or multiple founder events.

In the Iberian Peninsula, Roma groups were well-accepted at their arrival, but at the end of the fifteenth century, with the unification of Castile and Aragon crowns, the nomad Roma groups were forced to become sedentary and suffered continuous persecutions [1]. As we remark, the present-day Iberian Roma exhibit high levels of non-Roma European ancestry, with an admixture event estimated around 1250–1600. Although GLOBETROTTER did not infer two independent admixture events as might be expected in the Iberian Roma, two different European footprints are identified: the Balkan and the non-Roma Iberian components. The detection of a single signal of admixture could be explained by a rapid expansion from the Balkans to the Iberian Peninsula, with a short time gap between the two events, or due to continuous gene flow between non-Roma Europeans and Roma groups during their migration within Europe. In fact, if the time ranges between two events are close, the ability of GLOBETROTTER to distinguish between two admixture pulses from a single pulse decreases [31].

Besides between-country heterogeneity, the present study further identifies within-country Roma substructure in the Iberian Peninsula, partially correlated with geography: two clusters are restricted to the northwestern and central part of the peninsula (IberianRoma-1 and IberianRoma-2), another cluster mainly represents Roma samples from the south (IberianRoma-3) and the last one contains all the northeastern individuals (IberianRoma-4). These groups differ both in ancestry proportions and inbreeding levels, which can be the result of different demographic patterns, as the different laws concerning the Roma people in the Iberian Peninsula were neither homogeneous nor permanent [1]. As mentioned above, IberianRoma-4 is the most differentiated cluster. It exhibits more non-Roma Iberian ancestry, the inferred date of the admixture event is the most recent one (1532–1730), and it presents the lowest inbreeding levels. Altogether this can be explained by the extensive admixture with the non-Roma Iberian population. In fact, historical records confirm that both nomadic and sedentary Roma groups in the Principality of Catalonia were highly linked and interrelated with the non-Roma society [36]. In addition, their European ancestral source contains groups from North Italy and Northwestern Europe that are absent in the rest of Iberian Roma samples, which might point to either a posterior arrival to the Iberian Peninsula after admixing with these European populations or due to the constant movement of Roma groups between Southeastern France and Northeastern Spain [36]. The Iberian group representing the most southern location, IberianRoma-3, has a genetic particularity: it has around 1% of Northwest African ancestry, which probably corresponds to the North African admixture found in the southern and western parts of the Iberian Peninsula, during the Arab expansion (711–1248) [28,29]. The fact that the North African component is only found in IberianRoma-3 samples, who also show Balkan ancestry, contributes to reject the hypothesis of a Roma migration route to Iberia from North Africa [30]. IberianRoma-1 has more non-Roma Iberian component than IberianRoma-2, although these two clusters contain samples from the same region. These results highlight that, even within Roma groups who live in the same geographic region, distinct social dynamics (ie. itinerant vs sedentary lifestyles) caused the application of different laws that might have shaped their current genetic landscape. On the contrary, some geographical patterns have probably been diluted due to the continuous movement and admixture among Roma groups, especially after 1749 with the general imprisonment of Spanish Romani, who were captured and relocated, although the effects of this event were not uniform throughout the Roma community, enabling the identification of present-day geographical patterns within Iberia Roma [37].

The present study attempts to characterize the European Roma and describe their South Asian and West Eurasian components using fine-scale methods. On the one hand, we have targeted the putative South Asian ancestry of the Roma in a specific group of Punjabi and Southeastern Indian individuals, representing a small group of proto-Roma founders with low levels of the West Eurasian ancestry. Besides, our results show that the recent West Eurasian component (around 65% of the Roma genomes) was acquired between 1270–1580, during the Roma diaspora. Specifically, we have detected and characterized the Balkan genetic footprint in all European Roma groups and the Baltic and Iberian components in the Northern and Western Roma groups, respectively, likely due to a continuous non-Roma gene flow during their dispersal through Europe. On the other hand, we have found genetic substructure within the Iberian Roma, with different groups and different levels of non-Roma admixture, as a result of the complex historical events occurred in the Peninsula. Further studies are needed to fully understand the genetic substructure of the Roma population as well as to provide new insights into the migration routes undertaken by the European Roma shaping their current genetic landscape. The use of migration group data (Balkan, Romungro and Vlax group assignation) would add an additional layer of information in both genome-wide and complete uniparental markers analyses, as it has been suggested that Roma genetic diversity might be primarily structured by migration route [11,12].

## Materials and methods

### Ethics statement

Written informed consent was obtained from all the volunteers and the present project has the corresponding IRB approval (CEIC-Parc de Salut Mar 2016/6723/I).

### Samples and genotyping

#### Dataset1

The present study is based on 152 previously published European Roma genome-wide data from Bulgaria, Croatia, Estonia, Greece, Hungary, Lithuania, Portugal, Romania, Serbia, Spain, Slovakia, Ukraine, and Wales, genotyped with Affymetrix 6.0 platform [15]. As non-Roma reference samples, the present dataset includes previously published whole genome sequences and genotyping data from Europe, Middle East, Caucasus, Africa, and Central and South Asia [38–41]. Populations from the reference dataset were normalized to 20 individuals to minimize possible biases as a result of different sample sizes, except for Punjabis from Lahore (PJL) and Iberian population in Spain (IBS) from 1000G [38], due to their pivotal relevance for the goals of the present project. Missing SNPs in more than 10% of the individuals or with a minor allele frequency (MAF) below 0.01 were removed, and individuals with more than 10% of missing calls or sharing more than 85% of identity by state (IBS) values were removed. We applied the same quality control filters both in the autosomes and the X chromosome. The final Dataset1 with the European Roma and the non-Roma reference samples includes 324,075 autosomal SNPs and 23,182 X chromosome SNPs in 952 individuals (S1 Table).

#### Dataset2

In addition, the present study uses 34 newly genotyped Iberian Roma samples, from Romani self-defined volunteers residing in Barcelona, Bilbao, Granada, Madrid, and Porto areas, whose four grandparents were born in these regions. Blood and saliva samples were used to extract the DNA, which was genotyped with the Affymetrix Axiom Genome-Wide Human Origins 1 array, and genotype calling was performed with the Axiom Analysis Suite 2.0 software using standard parameters. A standard quality control protocol was applied with PLINK 1.9 [42] in order to filter out genotyping errors: missing SNPs in more than 10% of the individuals, individuals with more than 10% of missing calls, SNPs failing Hardy-Weinberg exact test at 0.05 significance threshold, and SNPs with a MAF below 0.01. In addition, to avoid possible relationships among individuals, samples sharing more than 85% of IBS values were removed. Data is available in https://figshare.com/articles/Iberian_Roma_dataset/7594730. Previously published whole genome sequences and genotyping data were merged with our Iberian Romani dataset, including samples from Europe, Middle East, Caucasus, Africa, and Central and South Asia [38,40,41,43]. As in Dataset1, reference non-Roma populations were normalized to 20 individuals to minimize possible biases, except PJL and IBS samples from 1000G [38]. The final Dataset2 with the Iberian Roma and the reference samples includes 360,676 SNPs and 1,333 individuals (S9 Table).

In order to keep a high density of SNPs, we did not merge Dataset1 with Dataset2, instead, we performed all the analyses separately, as they were genotyped with two different array platforms.

### Population structure analyses

A linkage disequilibrium pruning was performed for the analyses that require it using PLINK 1.9 [42] with standard parameters (window size of 50 SNPs, 5 SNPs shift at each step, and an r2 threshold of 0.5) in both Dataset1 and Dataset2, leaving 192,815 and 186,374 SNPs, respectively.

In order to examine the Roma population structure in a worldwide context, a PCA was performed with SmartPCA program implemented in EIGENSOFT 4.2 package [44], and 20 runs of ADMIXTURE [45] with different random seed tests were computed for different ancestral components (k = 2 to 8). We used pong [46] to identify and visualize modal ADMIXTURE results for each value of K. Both analyses were performed in Dataset1 and Dataset2 independently.

### Fine-scale population genetic characterization (ChromoPainter and fineSTRUCTURE)

The phasing of the Dataset1 and Dataset2 autosomal data was performed, independently, with SHAPEIT [47], using the population-averaged genetic map from the HapMap phase II [48] and the 1000G dataset as a reference panel [38].

ChromoPainter [21], based on a Hidden Markov Model (HMM) algorithm, aims to reconstruct the chromosome of each target individual (“recipient”) as a mosaic of haplotypes from the reference individuals (“donors”). This procedure is known as chromosome painting and their results can be summarized in a coancestry matrix, which shows for each recipient the total counts and length in cM of haplotypes that share a most recent common ancestor with each donor [21]. Intuitively, this matrix shows the haplotypes shared between each recipient and each donor individual. First, in order to infer the switch rate and global mutation probability (n and m parameters), ChromoPainter v2 was run in chromosomes 1, 7, 14, and 20, for 10 iterations of the expectation-maximization (EM) algorithm, painting each recipient (all individuals in the dataset) using all the donors (the rest of individuals in the dataset). For Dataset1, the inferred n and m parameter values were 251.11459 and 0.00023, respectively. Then, ChromoPainter v2 was run again in all chromosomes fixing these parameters. The total counts and lengths coancestry matrices were obtained by adding the matrices of all chromosomes.

FineSTRUCTURE [21] is an algorithm that infers the clustering of the samples considering the information in the ChromoPainter coancestry matrix. Using this clustering, it is possible to group the samples into genetically homogeneous clusters. First, fineSTRUCTURE was run for 2 million Markov Chain Monte Carlo (MCMC) iterations, sampling values every 10,000 iterations after 1 million “burn-in” iterations [49]. Then, fineSTRUCTURE was run again to perform 100,000 additional hill-climbing moves from the MCMC sample with the highest posterior probability to get the final cluster membership in a dendrogram format. This procedure was repeated three times and after comparing the consistency of the three dendrograms, we classified the 952 individuals from Dataset1 into 63 clusters, where the European Roma branch contains ten Roma clusters. The rest of Roma samples outside this clade (e.g. Welsh Roma) cluster with other European non-Roma samples, due to high levels of non-Roma European ancestry as described previously [15], thus they were removed for further analyses.

In order to estimate the copying profiles (i.e. average proportion of ancestry attributed to each donor group), ChromoPainter v2 was run in a different mode than described above: haplotype sharing was inferred between groups rather than independent individuals [49]. For this analysis all the individuals were grouped in the genetic clusters established according to fineSTRUCTURE where the ten European Romani clusters were settled as recipients and the rest of clusters as donors. In addition, we calculated the TVD metric as described in [49], which measures the differences between a pair of clusters (A, B) with copying vectors a and b and it can be calculated as:

$$\mathrm{TVD}(A,B)=0.5\times \sum_{i=1}^{n}\left(a_{i}‐b_{i}\right)$$ (1)

where n is the total number of donor groups. As suggested by Leslie S. et al [49], for each pair of clusters, individuals were randomly reassigned in one of the two clusters, and the new copying vectors a’ and b’, and the TVD values were recalculated for 1,000 permutations. P-values correspond to the proportion of permutations where TVD(A’,B’) > TVD(A,B) and reflect the strength of differences between the inferred pair of clusters. Corrected p-values were obtained after Bonferroni multiple test correction.

For Dataset2, the above procedures (ChromoPainter, fineSTRUCTURE, and TVD metric calculations) were also performed using the same approach, and the ChromoPainter switch rate and global mutation probability inferred using Dataset2 were 259.85269 and 0.00016, respectively. The fineSTRUCTURE dendrogram of Dataset2 was used to classify the 1,332 individuals into 88 clusters, where four of them belonged to Iberian Roma clusters. One Iberian Romani sample from Madrid (G32) was excluded, as it clustered with Iberian non-Roma samples, suggesting an extensive non-Roma ancestry.

We checked whether the ChromoPainter algorithm is able to correctly distinguish between the two sources of West Eurasian ancestry in the Roma population, in order to avoid misleading results when inferring the admixture sources: the AWE component (pre-exodus from India) as South Asian ancestry, and the recent West Eurasian admixture (post-exodus from India) as West Eurasian (see S1 Note, S5 and S6 Figs, S4 Table).

### Inferring admixture events with GLOBETROTTER

GLOBETROTTER [31] is a method designed to characterize and date admixture events between source populations (which are a composite of surrogate populations) that have shaped the genetic history of a target population. The dating estimation is based on the principle that the size of the haplotypes decreases over successive generations due to recombination. GLOBETROTTER algorithm uses the haplotype sharing results from ChromoPainter considering donor and recipients as groups of individuals. GLOBETROTTER was run for each of the ten Roma clusters in the European Roma branch from Dataset1 using ten painting samples per individual from ChromoPainter and the coancestry matrix of the genome-wide length of haplotype sharing. In order to identify the admixture events between source populations that have shaped the genetic history of European Roma, the surrogate populations included were all the European, Middle Eastern, Caucasian, and Asian clusters. The sample size of these clusters was normalized to a maximum of 21, which corresponds to the third quartile of all clusters sample sizes. First, in order to estimate p-values for evidence of admixture, GLOBETROTTER was run using the NULL procedure (standardize the coancestry curves by a “NULL” individual), with 100 bootstrap resamples. Then, GLOBETROTTER was run using the non-NULL inference to characterize the admixture events. These two GLOBETROTTER runs were checked for consistency. To estimate admixture date CIs, 100 bootstrap iterations were performed and a generation time of 25 years was considered.

The same procedure was used to infer admixture events that have shaped the genetic history of the Iberian Roma from Dataset2. Thus, the target populations were the four Iberian Roma clusters, and the surrogate populations were all the European, North African, Middle Eastern, Caucasian, and Asian clusters. Spatial distributions of the major source proportions in each Iberian Roma cluster were computed in R using the kriging model in the package fields [50].

When describing the admixture sources that have shaped the Roma today, we use the term “non-Roma populations” to facilitate the understanding, although the admixture events occurred with “non-proto-Roma” groups.

### Characterizing South Asian origin of the proto-Roma

To further characterize the South Asian component of the Roma, we have estimated the proportion of WE ancestry in the South Asian clusters (ANI component) using f4 ratio estimation implemented in ADMIXTOOLS [51] as: $\alpha =\frac{f_{4}(\mathrm{YRI},\;\mathrm{Basque};\mathrm{India},\mathrm{Onge})}{f_{4}(\mathrm{YRI},\;\mathrm{Basque};\mathrm{Georgians},\mathrm{Onge})}$ [20], computing standard error with a Block Jackknife with a block size of 5cM. For this analysis, we have included Onge samples from [52]. We have calculated the ANI proportion in the Roma groups from the relative contribution (inferred by GLOBETROTTER) of each South Asian cluster.

### Testing sex-biased gene flow through ancestry proportion differences between X chromosome and autosomes

The X chromosome from Dataset1 was phased using the same parameters as the autosomes, as described previously [39]; and ChromoPainter v2 [21] was run with all European Roma samples as recipients and the non-Roma European, Middle East, Caucasus, and South Asian clusters as donors using only the X chromosome. Then, the ancestry profiles of the X chromosome were estimated for each individual in each Roma cluster by applying SOURCEFIND, a new Bayesian model-based approach [53], with 200,000 MCMC samples, sampling every 1,000 iterations. Once we obtained the estimated proportions of each donor cluster in the X chromosome of the Roma from the MCM sample with the highest posterior probability, we summed them to get the European, MiddleEast-Caucasus, and South Asian proportions that contribute to the Roma ancestry. The same procedure was applied to the autosomes. To test for sex-biased gene flow in the Roma samples, we obtained the ancestry differences per individual by subtracting the European, MiddleEast-Caucasus, and South Asian proportions between the autosomes and the X chromosome grouping all Roma individuals together. A Wilcoxon signed-rank test across individuals between the autosomes and the X chromosome was applied to obtain a p-value of the differences, with Bonferroni correction. In addition, we tested the European ancestry differences for each Roma cluster. To avoid possible biases due to different number of SNPs, we not only compared the whole set of autosomes against the X chromosome, but also each autosome separately against the X chromosome (see S2 Note, S7 Fig, S6 Table).

### Inbreeding analyses and Ne estimation

ROH analyses were performed to assess the inbreeding levels among the Roma groups. ROH segments were identified using PLINK 1.9 [42], considering ROH with at least 50 SNPs of length 500 kb and a maximum gap between a pair of consecutive SNPs of 100 kb, as these parameters account for locally low SNP density in SNP arrays [54]. For comparative purposes, Dataset1 analysis included two clusters with putative higher levels of inbreeding, from Europe (Basque) and from South Asia (Kalash); and two with low levels, from Europe (Balkan-2) and from South Asia (Punjabi-1). For Dataset2, we included Basque and Kalash clusters, and SW-Europe2 and Punjabi-1.

Changes in Ne through generations were estimated for the Roma groups from IBD segments. The Roma samples belong to an admixed population, and thus, in order to detect IBD segments, we applied RefinedIBD [55], a haplotype-based method, with default parameters; and merged the segments with gaps to avoid the underestimation of segment lengths [56]. Then, using these IBD segments and the HapMap GRCh37 genetic map [48], IBDNe [57] was run with default parameters to infer Ne estimates with 95% CIs at each generation, assuming 25 years per generation. Although these methods were first designed to deal with sequence data, this approach applied to genome-wide array data has a high confidence in recent periods (from present to around 50 generations ago) [57]. For Dataset1, the analysis was performed on the ten European Roma clusters and the reference cluster NorthItaly. For Dataset2, it was performed on the four Iberian Roma clusters and SW-Europe2 as reference. In addition, we checked whether the Ne estimations correlate with the admixture event detected with GLOBETROTTER in each Roma group, regarding both the proportion of West Eurasian source and the admixture dates (see S3A Note).

Finally, we estimated the Ne of the ancestral Roma populations, following the same procedure as in Browning et al. [56], to compare the ancestry-specific Ne of the European, MiddleEast-Caucasian and South Asian sources prior to the admixture, grouping all Roma samples together (as we assume that the Roma groups split after the arrival to Europe). First, we performed a local ancestry inference (LAI) with RFMix v1.5.4 [58], using as sources the donor populations identified in the GLOBETROTTER analysis, grouped in three categories: Europe, MiddleEast-Caucasus and South Asia. Although Europe and MiddleEast-Caucasus ancestries are similar, Xue et al. [59] showed that RFMix is able to accurately infer local ancestry segments, using balanced reference panels with key features comparable to our study (e.g. SNP array data and admixture sources). After checking the correlation between the ancestry proportions of RFMix and GLOBETROTTER (see S3B Note, S19 Fig), we followed Browning et al. [56] pipeline: rephasing of the RFMix output, filtering of the IBD segments by ancestry and calculation of the ancestry-adjustment number of pairs of sampled haplotypes. Then, IBDNe [57] was run with default parameters to infer ancestry-specific Ne estimates with 95% CIs at each generation, assuming 25 years per generation. Finally, we calculated the fold-change of the Ne CIs between the three ancestral populations, one generation before the start of the admixture (i.e. lowest CI inferred from GLOBETROTTER) and compared it with the fold-change between the current ancestry proportions inferred with GLOBETROTTER.

## Supporting information

S1 Note Two sources of West Eurasian ancestry in the Roma population. (DOCX)

S2 Note X chromosome and autosomes ancestry comparisons. (DOCX)

S3 Note Correlations between Ne estimates and admixture events inferred from GLOBETROTTER. A. Ne estimation for each European Roma group. B. Ancestry-specific Ne estimation.
(DOCX)

S4 Note Comparison between ancestry-specific Ne and ancestry proportions in the Roma groups inferred with GLOBETROTTER. (DOCX)

S1 Fig PCA with samples from Europe, Africa, Middle East, Caucasus, Central and South Asia, and European Roma (black border) (Dataset 1). (PDF)

S2 Fig ADMIXTURE analysis for k = 2 to 8 ancestral components using samples from Europe, Africa, Middle East, Caucasus, Central and South Asia, and European Roma (Dataset 1). Each vertical line represents one individual and each color represents the proportion of each ancestral component. Major modes are shown in A and minor modes in B.
(PDF)

S3 Fig fineSTRUCTURE dendrogram with 952 samples, reflecting the five super-groups (Europe, MiddleEast-Caucasus, European Roma, Central-SouthAsia, and MiddleEast-Africa) (Dataset 1). Colored boxes include clusters from the European Roma branch and those non-Roma clusters identified as contributing sources to the Roma genomes in the GLOBETROTTER results.
(PDF)

S4 Fig Total Variance Distance (TVD) values for each pairwise comparison between the ten European Roma clusters (Dataset 1). (PDF)

S5 Fig Notched boxplots (showing the median, 95% confidence interval of the median, 25^th^ and 75^th^ percentiles across indiviuals from each cluster) of the chunklengths given by all West Eurasian donor populations to each Indian cluster inferred by ChromoPainter (Dataset 1). (PDF)

S6 Fig Density plots of the chunklegths (A) and chunkcounts (B) given by all West Eurasian donor populations to the European Roma, when using all Indian clusters as South Asian donors (in blue) and when using only NE-India2 as South Asian donor (in black). C. Density plot with, in black, the overlapping segments between the two analyses (using all Indian clusters and using only NE-India2) (median = 442569) and, in blue, the distribution of those segments found only when using only NE-India2 (median = 92252) (Dataset 1).
(PDF)

S7 Fig A. Density distributions of European, MiddleEast-Caucasus, and South Asian ancestry differences between the autosomes (whole set of autosomes) and X chromosome (estimated through SOURCEFIND method) grouping of Roma samples together. B. Density distributions of European ancestry differences between the autosomes (whole set of autosomes) and X chromosome for each Roma cluster. Positive values indicate higher ancestry proportions in the autosomes than in the X chromosome, while negative values indicate higher ancestry proportions in the X chromosome than in the autosomes. (Dataset 1).
(PDF)

S8 Fig Effective population size changes in log scale through time with 95% confidence intervals for each European Roma cluster, with NorthItaly cluster as a reference (Dataset 1). X-axis corresponds to number of generations ago. The vertical dotted lines represent the start of the admixture in each group (lowerCI of the admixture date inferred with GLOBETROTTER).
(PDF)

S9 Fig Correlations between the admixture events inferred with GLOBETROTTER and the Ne estimates. Each dot represents a Roma cluster (Dataset 1). A. Correlation between the start of the admixture (lower CI date in generations ago) and the inflection time (in generations ago) from the upper CI Ne (i) and from the lower CI Ne (ii). B. Correlation between the proportion of the GLOBETROTTER major source (West Eurasian proportion) and inferred current Ne (at g_0_) from the upper CI Ne (i) and from the lower CI Ne (ii). C. Correlation between the proportion of the GLOBETROTTER major source (West Eurasian proportion) and the slope after the “inflection time” calculated from the upper CI Ne (i) and the lower CI Ne (ii).
(PDF)

S10 Fig Mean total length of each individual genome in runs of homozygosity in each ROH category (from 1–2 to 9–10 Mb) (Dataset 1). Each vertical bar represents a population group: reference populations (greyish colors) and European Roma clusters.
(PDF)

S11 Fig PCA with samples from Europe, North Africa, Middle East, Caucasus, Central and South Asia, and Iberian Romani (black border) (Dataset 2). (PDF)

S12 Fig ADMIXTURE analysis for k = 2 to 8 ancestral components with samples from Europe, North Africa, Middle East, Caucasus, Central and South Asia, and Iberian Romani (Dataset 2). Each vertical line represents one individual and each color represents the proportion of each ancestral component. Major modes are shown in A and minor modes in B.
(PDF)

S13 Fig fineSTRUCTURE dendrogram with 1,332 samples, reflecting the five super-groups (MiddleEast-Africa, Europe, Iberian Roma, MiddleEast-Caucasus, and Central-SouthAsia) and the Iberian Roma clusters (Dataset 2). Colored boxes include Iberian Roma clusters and those non-Roma clusters identified as contributing sources to the Roma genomes in the GLOBETROTTER results.
(PDF)

S14 Fig Iberian Roma substructure and West Eurasian ancestry (Dataset 2). **(**A) Iberian Roma sampled groups (pie charts are colored according the clusters in B) and their West Eurasian donors in the GLOBETROTTER analysis. (B) Iberian Roma fineSTRUCTURE dendrogam showing the four Iberian Roma clusters. (C) Major source of the admixture event inferred by GLOBETROTTER: for each Roma cluster, the proportion (in percentage) of the major source and a horizontal bar with the proportions of each donor populations (colored as in A), that contribute a minimum of 0.2 to the major source, are shown.
(PDF)

S15 Fig TVD values for each pairwise comparison between the four Iberian Roma clusters (Dataset 2). (PDF)

S16 Fig Density plots showing the GLOBETROTTER estimations of admixture dates with 100 bootstrap iterations for each Iberian Roma cluster (Dataset 2). Admixture dates (x-axis) are shown in years CE (assuming a generation time of 25 years) and in generations agp (GA).
(PDF)

S17 Fig Mean total length of each individual genome in runs of homozygosity in each ROH category (from 1–2 to 9–10 Mb) (Dataset 2). Each vertical bar represents a population group: reference populations (greyish colors) and Iberian Roma clusters (greenish colors).
(PDF)

S18 Fig Effective population size changes in log scale through time with 95% confidence intervals for each Iberian Roma cluster and SW-Europe2, as a reference (Dataset 2). X-axis corresponds to number of generations ago. The vertical dotted lines represent the start of the admixture in each group (lowerCI of the admixture date inferred with GLOBETROTTER).
(PDF)

S19 Fig Correlation between the West Eurasian proportion inferred from GLOBETROTTER and the West Eurasian proportion from RFMix LAI. Each dot represents a Roma cluster (Dataset 1). The dashed line represents the line of equality (x = y).
(PDF)

S1 Table Sample dataset including information of population, cluster classification, sex (for Roma samples) and reference (Dastaset1). (XLSX)

S2 Table Total Variance Distance and p-values between pairs of European Roma clusters (Dastaset1), after Bonferroni correction. (XLSX)

S3 Table GLOBETROTTER results for each European Roma cluster (Dastaset1), describing type of admixture (and its measures of “goodness-of-fit”), mean date and CI 95% and composition of the major and minor sources. (XLSX)

S4 Table p-values resulting from Wilcoxon test of West Eurasian haplotype lengths for each pair of Indian clusters (after Bonferroni correction) (Dastaset1). (XLSX)

S5 Table A) Proportions of ANI component in South Asian clusters (ANI estimates from f4 ratio results with standard error and Z scores). B) Proportions of AWE component in European Roma clusters, estimated from the relative contribution of each South Asian cluster to the Roma genomes inferred by GLOBETROTTER.
(XLSX)

S6 Table A) Number of snps per chromosome. B) Difference of ancestry proportions (European, MiddleEastern-Caucasus, South Asian), between the autosomes and the X chromosome grouping of Roma samples together. Positive values indicate higher ancestry proportions in the autosomes than in the X chromosome, while negative values indicate higher ancestry proportions in the X chromosome than in the autosomes. Standard deviations and Bonferroni corrected p-value (p < 0.05 *; p < 0.01 **; p < 0.001 ***) are shown in brackets C) Difference of European ancestry proportions between the autosomes and the X chromosome. Positive values indicate higher ancestry proportions in the autosomes than in the X chromosome, while negative values indicate higher ancestry proportions in the X chromosome than in the autosomes. Standard deviations are shown in brackets. (Dastaset1).
(XLSX)

S7 Table A) Ne estimates and 95%CI for each ancestral-specific population at 34 generations ago. B) Fold change of the Ne 95%CI between the ancestral populations at 34 generations ago. C) Fold change of the ancestry proportions inferred with GLOBETROTTER. D) Fold change of the globel ancestry proportions inferred from the local ancestry inference with RFMix. (Dastaset1).
(XLSX)

S8 Table A) p-values resulting from Wilcoxon test for each pair of Roma clusters in each ROH category (after Bonferroni correction). p<0.05 are in bold. B) p-values resulting from Wilcoxon test for each pair of ROH categories in each Roma clusters (after FDR correction). p<0.05 are in bold. (Dastaset1).
(XLSX)

S9 Table Sample dataset including information of population, cluster classification and reference (Dastaset2). (XLSX)

S10 Table Total Variance Distance and p-values between pairs of Iberian Roma clusters (Dastaset2), after Bonferroni correction). (XLSX)

S11 Table GLOBETROTTER results for each Iberian Roma cluster (Dataset2), describing type of admixture (and its measures of “goodness-of-fit”), mean date and CI 95% and composition of the major and minor sources. (XLSX)

S12 Table A) p-values resulting from Wilcoxon test for each pair of Roma clusters in each ROH category (after Bonferroni correction). p<0.05 are in bold. B) p-values resulting from Wilcoxon test for each pair of ROH categories in each Roma clusters (after FDR correction). p<0.05 are in bold. (Dastaset2).
(XLSX)
