# Supplementary material for: European Roma groups show complex West Eurasian admixture footprints and a common South Asian genetic origin
Source: PLoS Genet. 2019 Sep 23;15(9):e1008417. doi: 10.1371/journal.pgen.1008417 (PMC6779411; doi:10.1371/journal.pgen.1008417)
Supplement: S1 Note — (DOCX) [file pgen.1008417.s001.docx]

# **S1 Note. Two sources of West Eurasian ancestry in the Roma population.**

The Roma population has to main sources of West Eurasian (WE) ancestry: the recent admixture during their diaspora with longer haplotypes (post-exodus from India) and the ancient West Eurasian (AWE) component derived from the South Asian populations with shorter haplotypes (pre-exodus from India) [1,2]. To check whether ChromoPainter [3] was able to distinguish between these two sources of WE ancestry in the Roma, we conducted the following analysis using Dataset1. First, we selected the South Asian cluster with significantly less WE component: NE-India2 (Fig. S5, Table S4). Second, setting the Roma as recipients, ChromoPainter was run independently twice: (i) using all European, Middle East, Caucasian and all South Asian groups as donors; and (ii) using all European, Middle East, and Caucasian donors, as in (i), but only NE-India2 as South Asian donor. Then, we calculated the haplotype lengths (Fig. S6A) and counts (Fig. S6B) shared between the Roma and the WE donors (i.e. excluding South Asian donors), in both ChromoPainter runs. The main objective of this procedure is to check whether the amount of haplotype sharing (in terms of both haplotype length and counts) between the Roma and West Eurasians changes depending on the South Asian proxy, which will give an idea about how ChromoPainter is assigning the two WE components of the Roma.

Following the above analysis, we found that the haplotype counts fold change between (ii) and (i) is significantly higher than the haplotype length fold change between (ii) and (i) (Wilcoxon signed-rank test across individuals p-value < 0.0001). In other words, the increase in WE haplotype counts between (ii) and (i) is higher than the haplotype length increase, meaning that more small chunks are assigned as WE in (ii) than in (i). In addition, we intersected those WE segments that overlap between analysis (i) and (ii), which might only include the recent WE component and we have subtracted only those WE segments found in (ii) not in (i), which might represent the ancestral WE that Roma carry from South Asia. From these sets of WE segments, we can observe two separate distributions of WE segment sizes (Fig. S6C). Thus, these results point that, when using only NE-India2 as South Asian donor, the AWE component of the Roma (shorter haplotypes) is assigned as WE, as it cannot be assigned as South Asian, because the NE-India2 group almost lacks this component. This suggests that when using all South Asian as donors, ChromoPainter demonstrates an ability to correctly distinguish between these two components: the recent WE admixture and the AWE from the South Asian populations.

1. Moorjani P, Patterson N, Loh P-R, Lipson M, Kisfali P, Melegh BI, et al. Reconstructing Roma history from genome-wide data. PLoS One. 2013;8(3):e58633.

2. Moorjani P, Thangaraj K, Patterson N, Lipson M, Loh P-R, Govindaraj P, et al. Genetic evidence for recent population mixture in India. Am J Hum Genet. 2013;93(3):422–38.

3. Lawson DJ, Hellenthal G, Myers S, Falush D. Inference of population structure using dense haplotype data. PLoS Genet. 2012;8(1):e1002453.
