# Supplementary material for: European Roma groups show complex West Eurasian admixture footprints and a common South Asian genetic origin
Source: PLoS Genet. 2019 Sep 23;15(9):e1008417. doi: 10.1371/journal.pgen.1008417 (PMC6779411; doi:10.1371/journal.pgen.1008417)
Supplement: S2 Note — (DOCX) [file pgen.1008417.s002.docx]

**S2 Note. X chromosome and autosomes ancestry comparisons.**

In the Materials and Methods section “Testing sex-biased gene flow through ancestry proportion differences between X chromosome and autosomes”, we have described how we have tested for sex-biased gene flow comparing the Roma X chromosome with the whole set of Roma autosomes. However, to avoid possible biases due to different number of SNPs (23,182 and 324,075, in the X chromosome and the whole set of autosomes, respectively), we performed the same analysis but comparing each autosome with the X chromosome separately (although chromosomes 13 to 22 contained less than 15,000 SNPs) to check whether the different number of SNPs could lead to spurious differences (Table S5A).

Thus, we checked the consistency between comparing the X chromosome with the whole set of autosomes or the X chromosome with each single autosome (Table S5B-C). For each Roma group and for each comparison (i.e. whole set of autosomes vs X chromosome; each autosome vs. X chromosome), we extracted three values: the mean and standard deviation of the difference and the Bonferroni corrected p-value (from the Wilcoxon test). These three estimates are consistent along all comparisons, showing the same patterns (Table S5B-C). This observation demonstrates that the ancestry differences between the autosomes and the X chromosome are not led by the different number of SNPs or stochastic oscillations of the ancestry proportions in each chromosome.
