# Supplementary material for: European Roma groups show complex West Eurasian admixture footprints and a common South Asian genetic origin
Source: PLoS Genet. 2019 Sep 23;15(9):e1008417. doi: 10.1371/journal.pgen.1008417 (PMC6779411; doi:10.1371/journal.pgen.1008417)
Supplement: S3 Note — A. Ne estimation for each European Roma group. B. Ancestry-specific Ne estimation. (DOCX) [file pgen.1008417.s003.docx]

**S3 Note. Correlations between Ne estimates and admixture events inferred from GLOBETROTTER.**

1. **Ne estimation for each European Roma group.**

We studied how the Ne history of the Roma is correlated with the amount of WE admixture by comparing two sets of parameters: three from the confidence intervals (CI) of the Ne estimates: (i) time when the Ne starts to increase or inflection time, (ii) current Ne (Ne at g_0_), and (iii) slope after the inflection time; and two from the GLOBETROTTER [1] inference: (i) start of the admixture event (lower CI of the admixture date) and (ii) proportion of WE (major source). We used the CIs of the Ne estimates to have two replicates in the correlation tests.

- Correlation between inflection time and start admixture. We define the inflection time as the time point when the Ne estimates start to increase after a long descending trend in the Roma groups (i.e. the date when the change in Ne switches sign). We identified the inflection times of the Ne CIs and applied a Spearman rank correlation, observing positive correlation coefficient values in both comparisons: 0.5829 (p-value < 0.07699) and 0.6505 (p-value < 0.04171) for upperCI Ne inflection time - lowerCI admixture date and lowerCI Ne inflection time - lowerCI admixture date, respectively (Fig S9A). Thus, the start of the admixture with West Eurasians and the consequent increase of the Ne are positively correlated.
- Correlation current Ne (Ne_0_) - % WE. We performed a Spearman rank correlation, observing a small non-significant rho in both comparisons: 0.3170 (p-value < 0.372) and 0.3780 (p-value < 0.2814) for upperCI Ne_0_ - %WE and lowerCI Ne_0_ - %WE, respectively (Fig S9B). Thus, the correlation between the Ne_0_ estimates and the % WE in the Roma groups is not statistically significant, although it could be due to the large CI of the Ne estimates.
- Correlation slope after inflection time - % WE (major source). We define the slope after the inflection time as the slope of the Ne curve between the inflection time and the current time (g_0_). Assuming a linear Ne curve between the inflection time and g_0_, we applied a linear regression to identify the slope (for both CI curves of the Ne), for all Roma groups, except RomaUkr whose slope is negative. Then, we applied a Spearman rank correlation, observing a small non-significant rho in both comparisons: 0.2762 (p-value < 0.472) and 0.2762 (p-value < 0.472) for upperCI slope - %WE and lowerCI slope - %WE, respectively (Fig S9C). Thus, the correlation between the slope after inflection time and the % WE in the Roma groups is not statistically significant.

We cannot rule out the possibility that the non-Roma admixture is affecting the estimates of Ne, leading to an observed increase (the last two tests might not be statistically significant due to the small sample size). However, we can argue that the observed Ne increase (due to the non-Roma admixture or a population expansion) is reflecting an increase of genetic diversity in the Roma groups.

1. **Ancestry-specific Ne estimation.**

Before estimating the ancestry-specific Ne, we checked the robustness of the LAI performed with the RFMix software [2], by testing the correlation between the ancestry proportions inferred with RFMix and GLOBETROTTER. To do so, we first calculated the global proportions from the RFMix LAI and added the Europe and MiddleEast-Caucasus components to estimate the global WE proportion for each Roma group. Thus, we compared the WE proportion from RFMix and the WE proportion from GLOBETROTTER (i.e. major source of the admixture event).

First, we applied the Shapiro-Wilk test to the RFMix WE proportion (p-value < 0.1375) and the GLOBETROTTER WE proportion (p-value < 0.01119) to test if the values are normally distributed. Then, we performed an F-test to compare the variances between the two WE proportions (p-value < 4.03e-16). As the GLOBETROTTER WE proportion deviates from normality and the ratio of variances is not equal, we performed a Spearman rank correlation, observing a significant positive rho of 0.9329 (p-value < 8.153e-05) (Fig S19). Thus, the WE proportions inferred with both methods (RFMix and GLOBETROTTER), are significantly correlated, meaning that the estimation of the ancestry-specific Ne will be based on robust ancestry proportions.

1. Hellenthal G, Busby GBJ, Band G, Wilson JF, Capelli C, Falush D, et al. A genetic atlas of human admixture history. Science. 2014;343(6172):747–51.

2. Maples BK, Gravel S, Kenny EE, Bustamante CD. RFMix : A Discriminative Modeling Approach for Rapid and Robust Local-Ancestry Inference. Am J Hum Genet. 2013;93(2):278–88.
