# Supplementary material for: European Roma groups show complex West Eurasian admixture footprints and a common South Asian genetic origin
Source: PLoS Genet. 2019 Sep 23;15(9):e1008417. doi: 10.1371/journal.pgen.1008417 (PMC6779411; doi:10.1371/journal.pgen.1008417)
Supplement: S4 Note — (DOCX) [file pgen.1008417.s004.docx]

**S4 Note. Comparison between ancestry-specific Ne and ancestry proportions in the Roma groups inferred with GLOBETROTTER.**

We compared the ancestry proportions inferred with GLOBETROTTER and the ancestry-specific Ne of the European, MiddleEast-Caucasus and South Asian source populations at 34 generations ago (more ancient lowest CI inferred from GLOBETROTTER is found in RomaMix-2 at 1164 CE). Although the inference accuracy of European vs MiddleEast-Caucasus segments can be lower than for cross-continental ancestries, we can observe the following general trends:

1. The fold change between the European Ne_g=34_ and the South Asian Ne_g=34_ (Table S6B) is higher than the fold change between the European ancestry proportion and the South Asian ancestry proportion (Table S6C-D)(except for those clusters with more WE admixture); meaning that the European Ne_g=34_ is higher than the European ancestry or/and that the South Asian Ne_g=34_ is lower than the South Asian ancestry.

$$\frac{\text{EUR} \text{Ne}_{\text{g=34}}}{\text{SASIAN} \text{Ne}_{\text{g=34}}}\text{ >} \frac{\text{\% EUR ancestry}}{\text{\% SASIAN ancestry}}$$

2. The fold change between the South Asian Ne_g=34_ and the MiddleEast-Caucasus Ne_g=34_ (Table S6B) is lower than the fold change between the South Asian ancestry proportion and the MiddleEast-Caucasus ancestry proportion (Table S6C-D); meaning that the MiddleEast-Caucasus Ne_g=34_ is higher than the MiddleEast-Caucasus ancestry or/and that the South Asian Ne_g=34_ is lower than the South Asian ancestry.

$$\frac{\text{SASIAN} \text{Ne}_{\text{g=34}}}{\text{ME\_C} \text{Ne}_{\text{g=34}}}\text{ <} \frac{\text{\% SASIAN ancestry}}{\text{\% ME\_C ancestry}}$$

When the number of ancestry-specific IBD segments is low, the coalescence probability is low and the ancestry-specific Ne is high [1], reflecting a high genetic diversity of the ancestral component. Thus, there are fewer European- and MiddleEast-Caucasus-specific IBD segments at g = 34 than the European and MiddleEast-Caucasus proportions, and more South Asian-specific IBD segments at g = 34 than the South Asian ancestry proportion in the Roma. These differences between the ancestry proportions and the ancestry-specific Ne could give some insights about the nature of the populations involved in the admixture event (the proto-Roma and the non-Roma populations): (a) the proto-Roma likely come from a small South Asian group that suffered a founder effect (a decrease in Ne led to an increase in South Asian-specific IBD segments, followed by a loss of genetic diversity); (b) the Middle Eastern and Caucasian ancestry might be derived from the admixture with multiple Middle Eastern and Caucasian non-related groups (with high Ne leading to fewer IBD segments and an increase in genetic diversity); and, in a similar way, (c) the European ancestry of the Roma population is due to the admixture with different non-Roma European populations.

Taking advantage of these results, we can infer some more features about the admixture event detected by GLOBETROTTER. The minor source is a mix of modern-day South Asian populations, but it is likely representing a unique, highly inbred ancestral proto-Roma population (not an amalgam of different ancestral groups who left India at the same moment). Whereas the fact that the major source is a mixture of West Eurasian populations does not mean that it was a unique ancestral source (as the South Asian one); instead, it might represent gene flow with different non-related European, Middle Eastern and Caucasian non-Roma populations.

1. Browning SR, Browning BL, Daviglus ML, Durazo RA, Schneiderman N, Kaplan RC, et al. Ancestry-specific recent effective population size in the Americas. PLoS Genet. 2018;14(5):e1007385.
