# Supplementary figures and images for: European Roma groups show complex West Eurasian admixture footprints and a common South Asian genetic origin
Source: PLoS Genet. 2019 Sep 23;15(9):e1008417. doi: 10.1371/journal.pgen.1008417 (PMC6779411; doi:10.1371/journal.pgen.1008417)

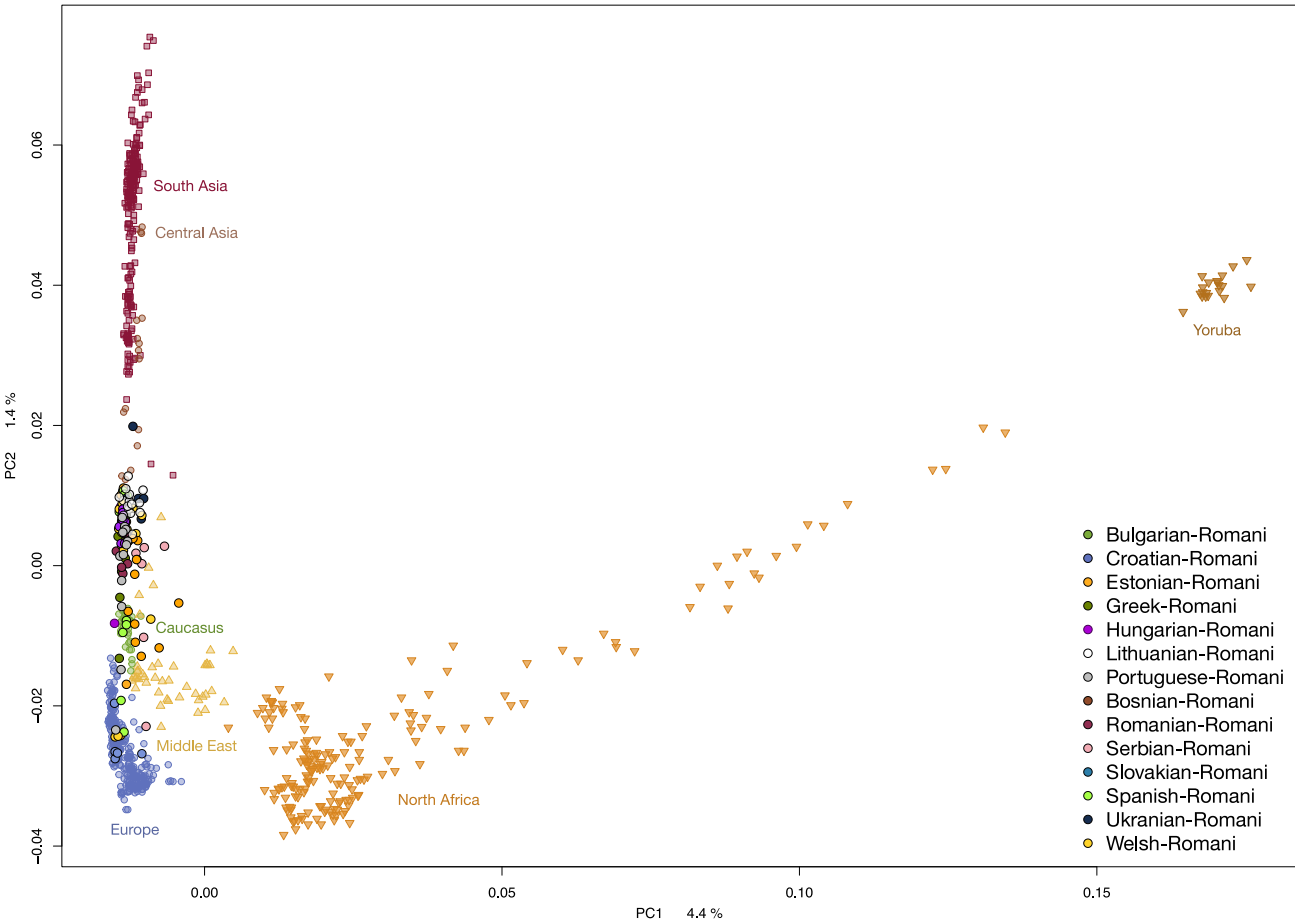

Supplement: S1 Fig — (PDF) [file pgen.1008417.s005.pdf]

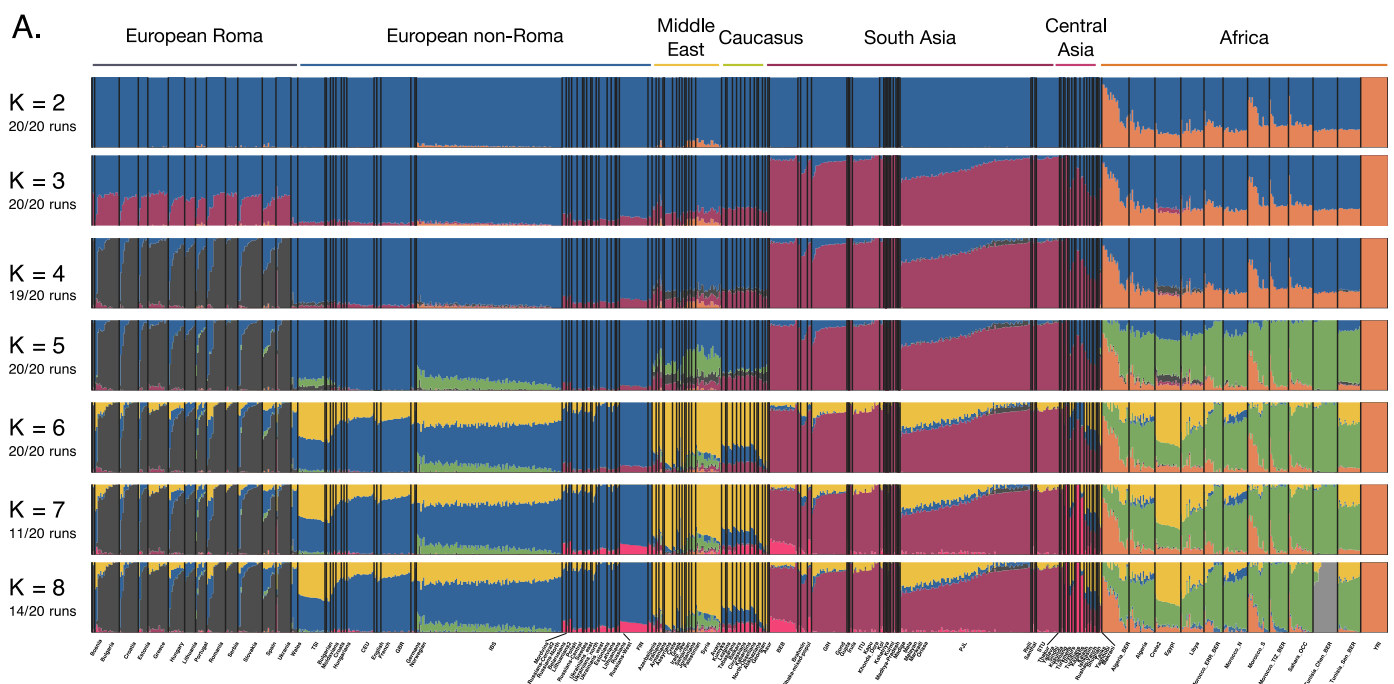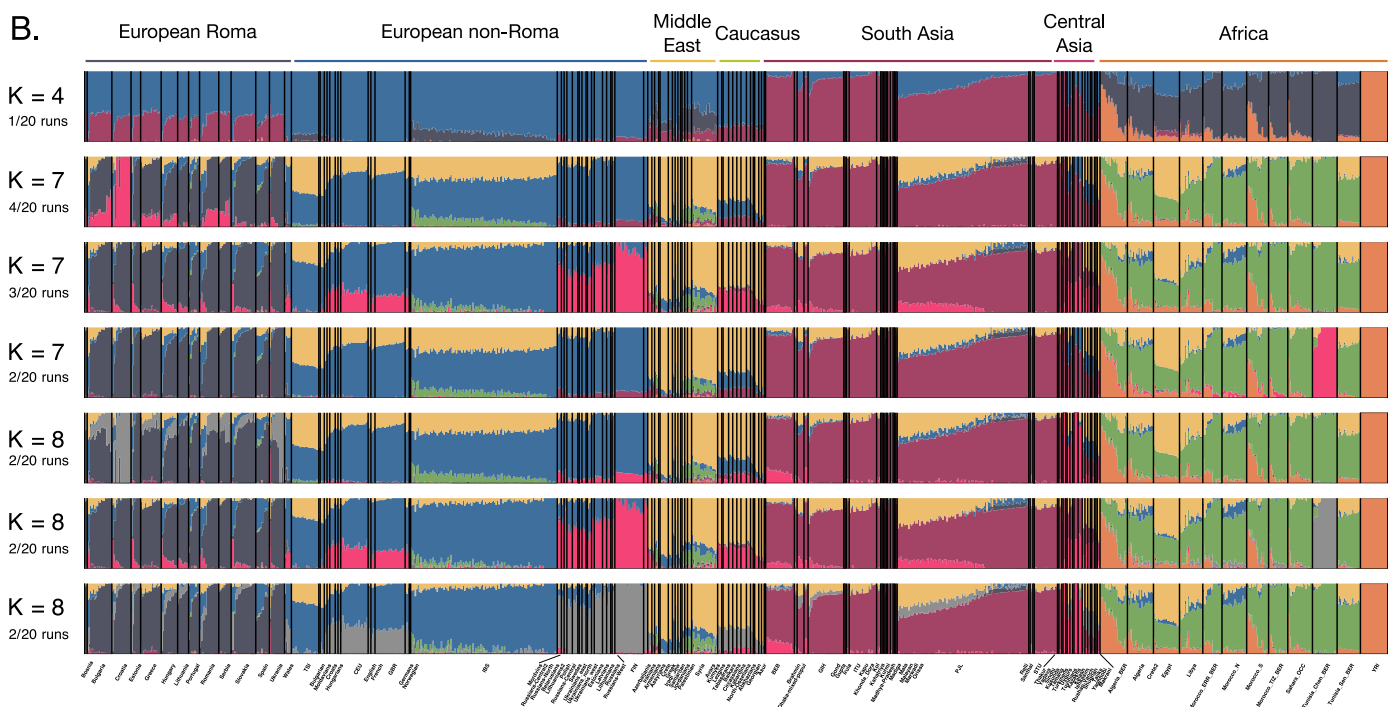

Supplement: S2 Fig — Each vertical line represents one individual and each color represents the proportion of each ancestral component. Major modes are shown in A and minor modes in B. (PDF) [file pgen.1008417.s006.pdf]

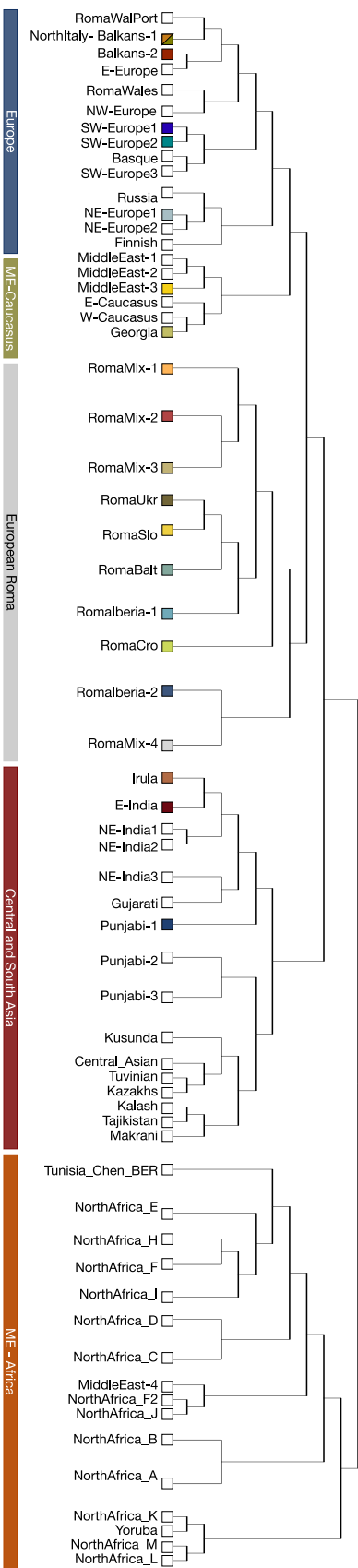

Supplement: S3 Fig — Colored boxes include clusters from the European Roma branch and those non-Roma clusters identified as contributing sources to the Roma genomes in the GLOBETROTTER results. (PDF) [file pgen.1008417.s007.pdf]

Chunklengths given by all West Eurasian donor populations

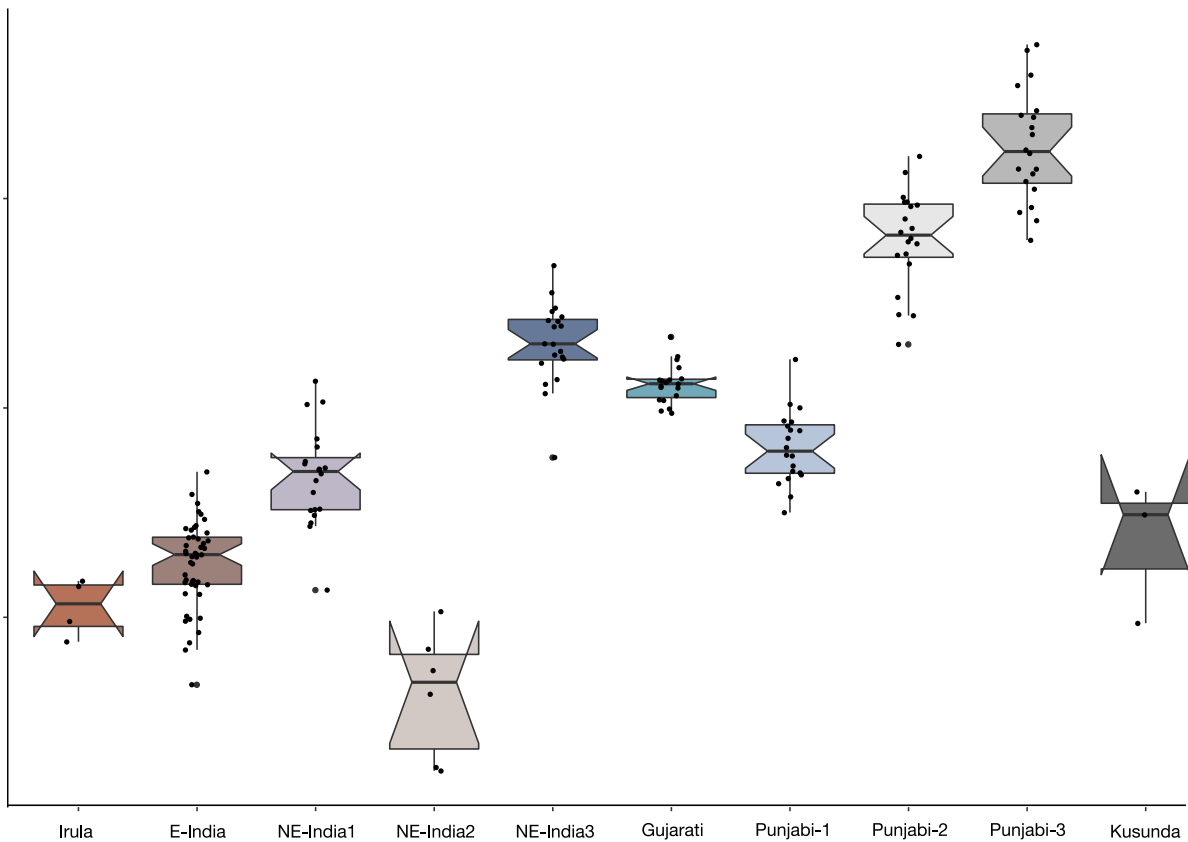

Supplement: S5 Fig — (PDF) [file pgen.1008417.s009.pdf]

A.

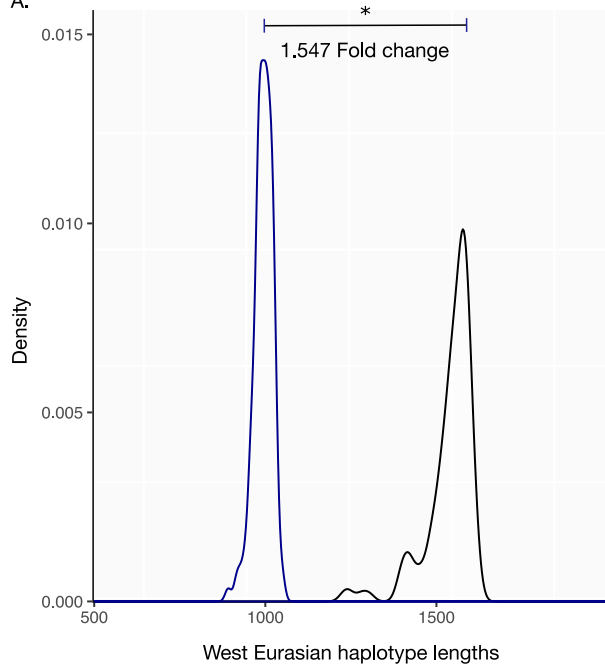

B.

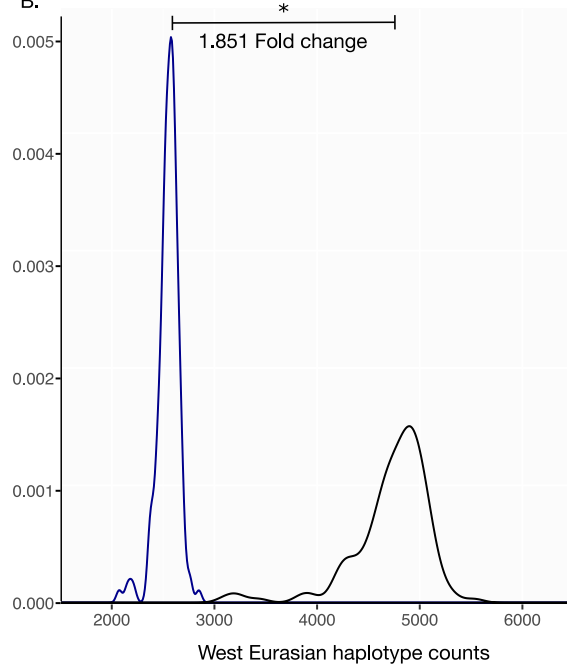

C.

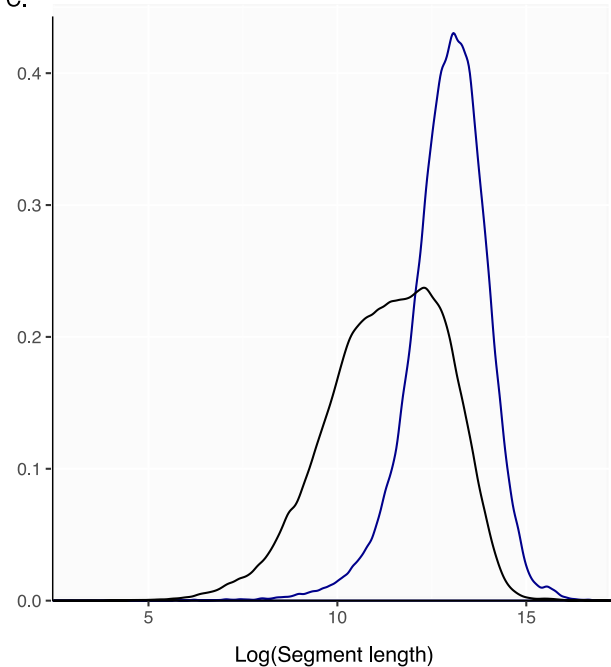

Supplement: S6 Fig — Density plots of the chunklegths (A) and chunkcounts (B) given by all West Eurasian donor populations to the European Roma, when using all Indian clusters as South Asian donors (in blue) and when using only NE-India2 as South Asian donor (in black). C. Density plot with, in black, the overlapping segments between the two analyses (using all Indian clusters and using only NE-India2) (median = 442569) and, in blue, the distribution of those segments found only when using only NE-India2 (median = 92252) (Dataset 1). (PDF) [file pgen.1008417.s010.pdf]

A.

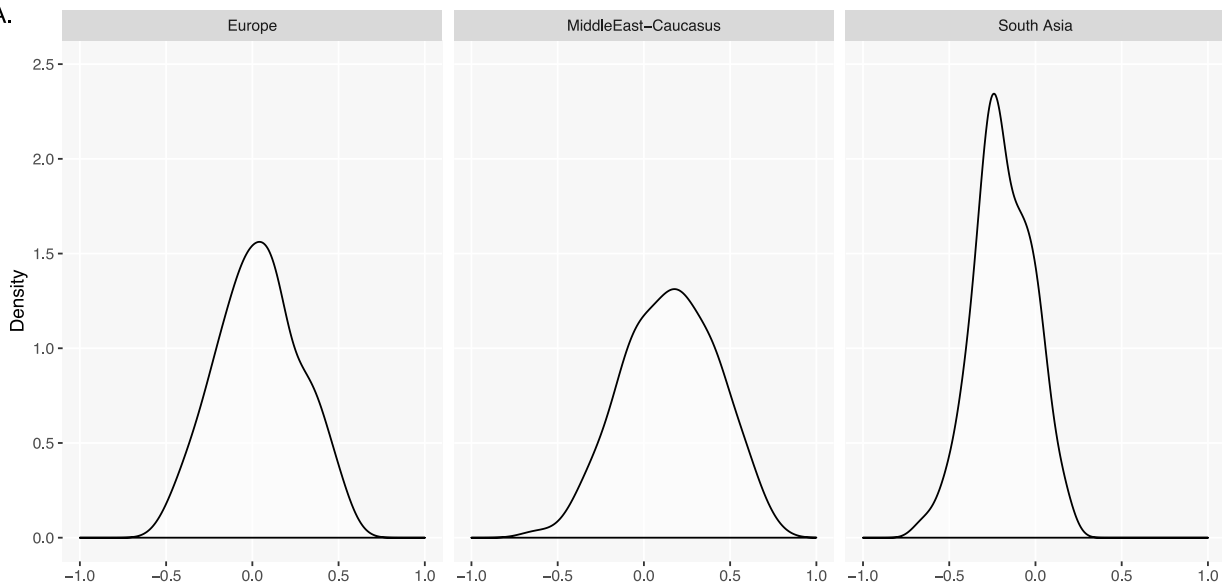

B.

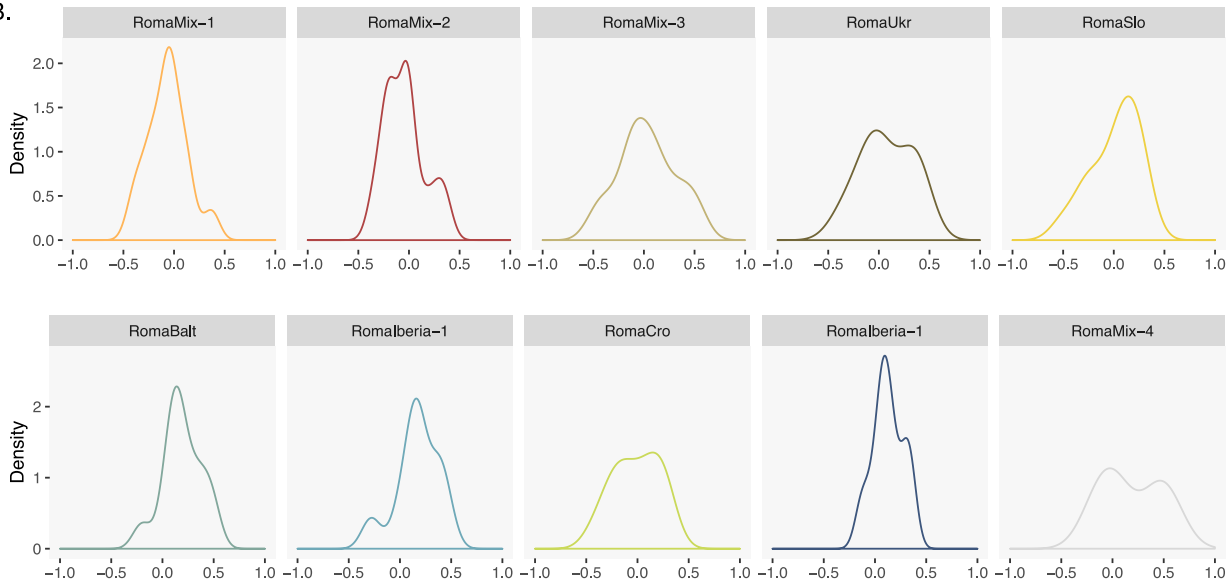

Supplement: S7 Fig — A. Density distributions of European, MiddleEast-Caucasus, and South Asian ancestry differences between the autosomes (whole set of autosomes) and X chromosome (estimated through SOURCEFIND method) grouping of Roma samples together. B. Density distributions of European ancestry differences between the autosomes (whole set of autosomes) and X chromosome for each Roma cluster. Positive values indicate higher ancestry proportions in the autosomes than in the X chromosome, while negative values indicate higher ancestry proportions in the X chromosome than in the autosomes. (Dataset 1). (PDF) [file pgen.1008417.s011.pdf]

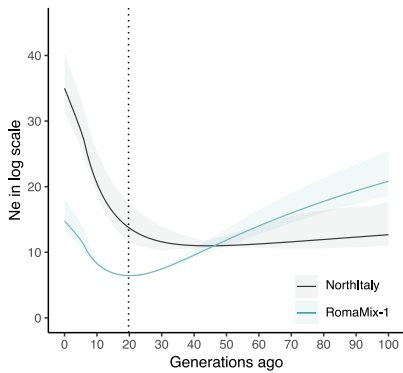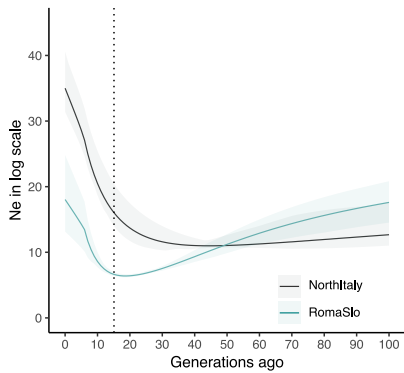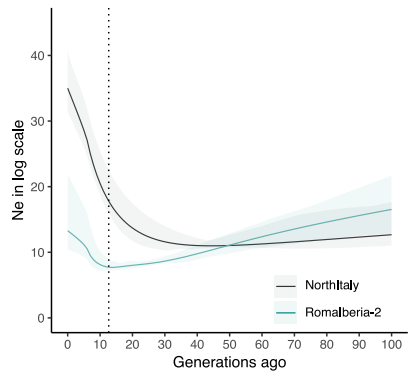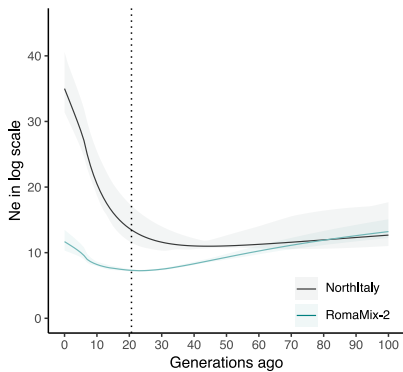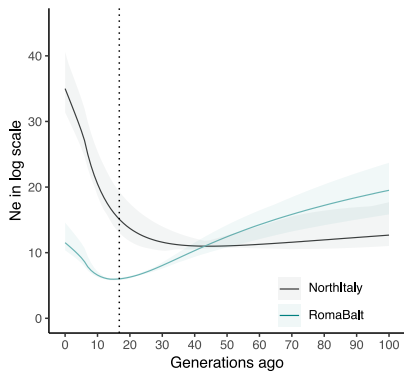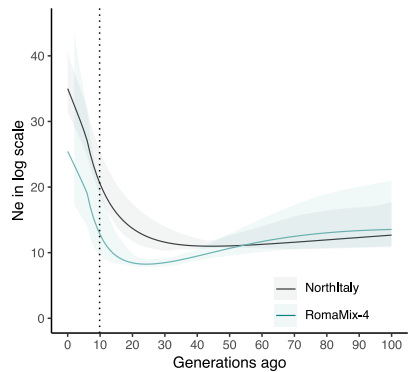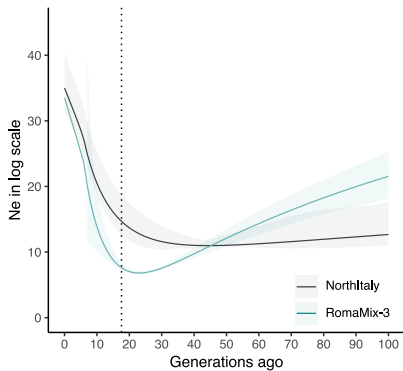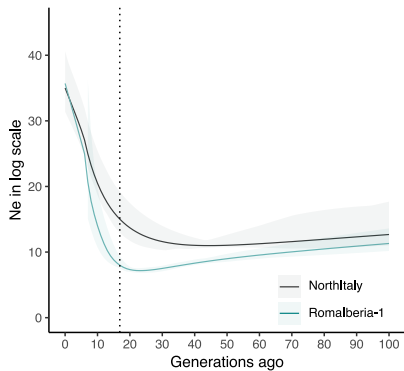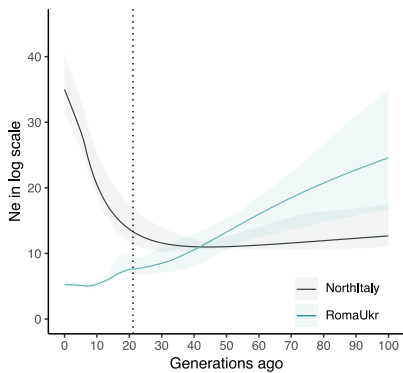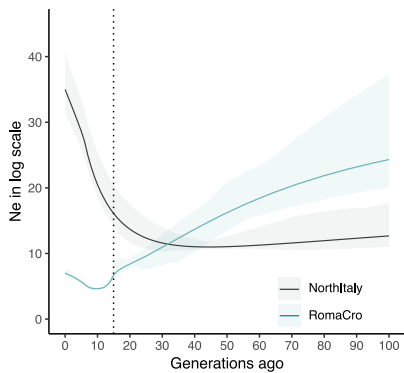

Supplement: S8 Fig — X-axis corresponds to number of generations ago. The vertical dotted lines represent the start of the admixture in each group (lowerCI of the admixture date inferred with GLOBETROTTER). (PDF) [file pgen.1008417.s012.pdf]

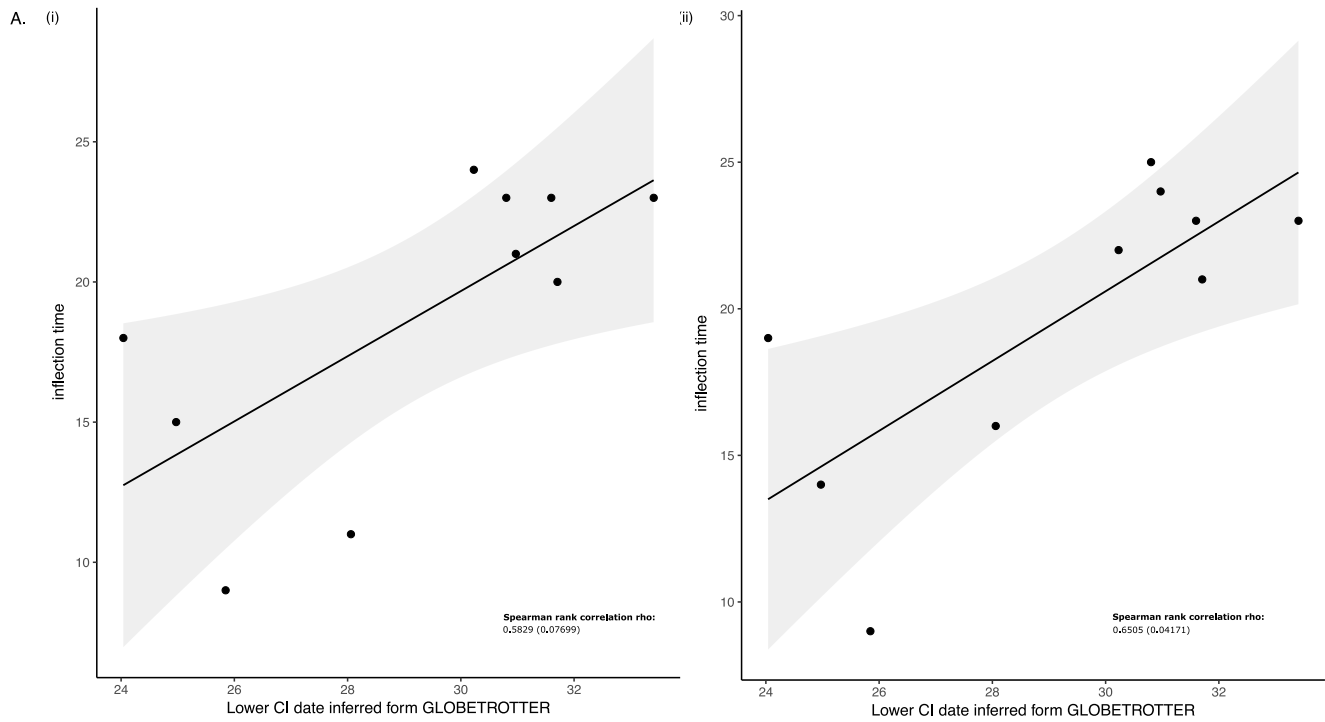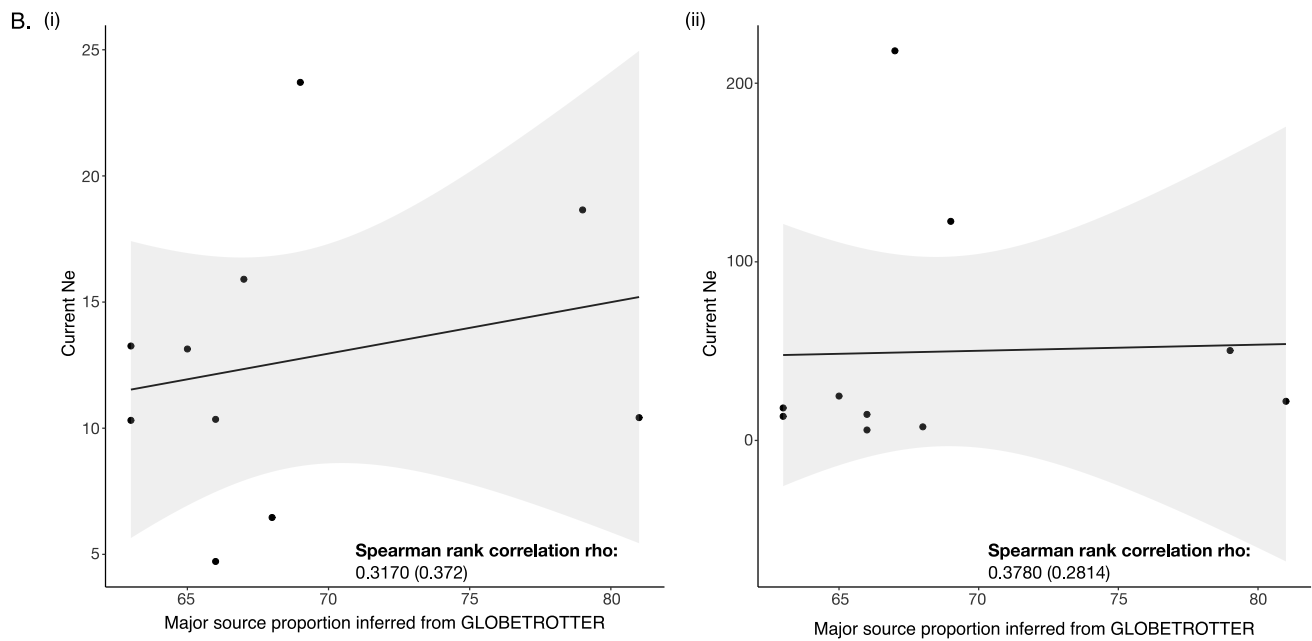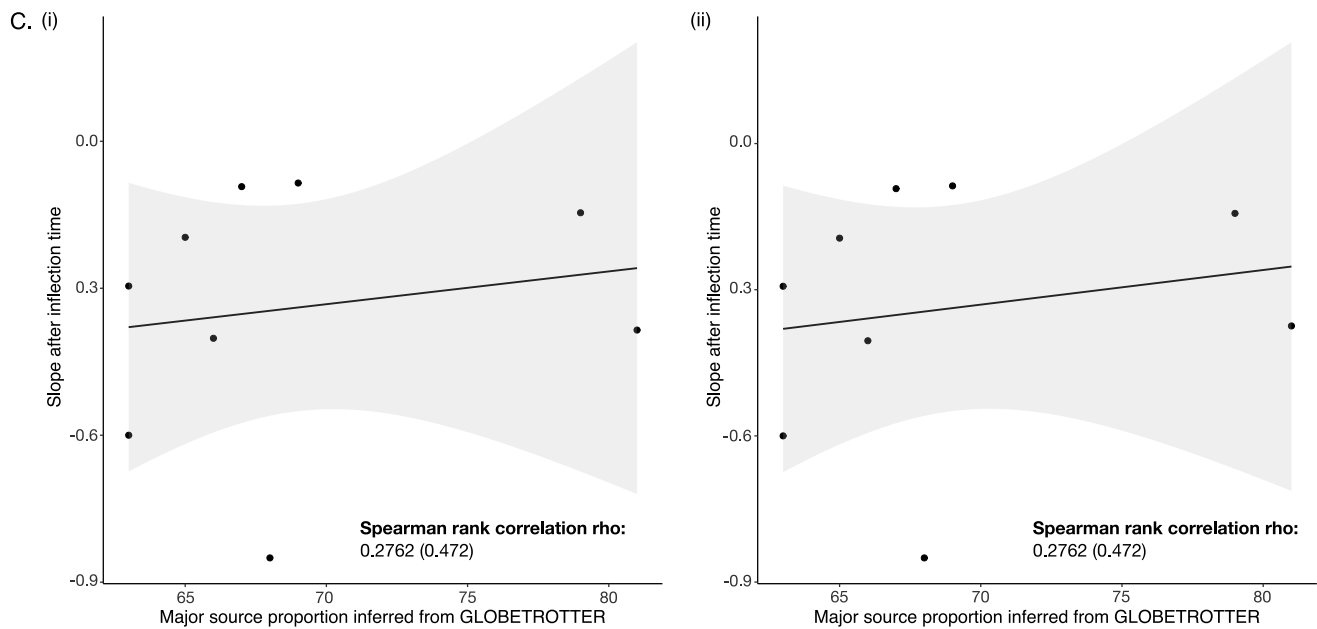

Supplement: S9 Fig — Each dot represents a Roma cluster (Dataset 1). A. Correlation between the start of the admixture (lower CI date in generations ago) and the inflection time (in generations ago) from the upper CI Ne (i) and from the lower CI Ne (ii). B. Correlation between the proportion of the GLOBETROTTER major source (West Eurasian proportion) and inferred current Ne (at g0) from the upper CI Ne (i) and from the lower CI Ne (ii). C. Correlation between the proportion of the GLOBETROTTER major source (West Eurasian proportion) and the slope after the “inflection time” calculated from the upper CI Ne (i) and the lower CI Ne (ii). (PDF) [file pgen.1008417.s013.pdf]

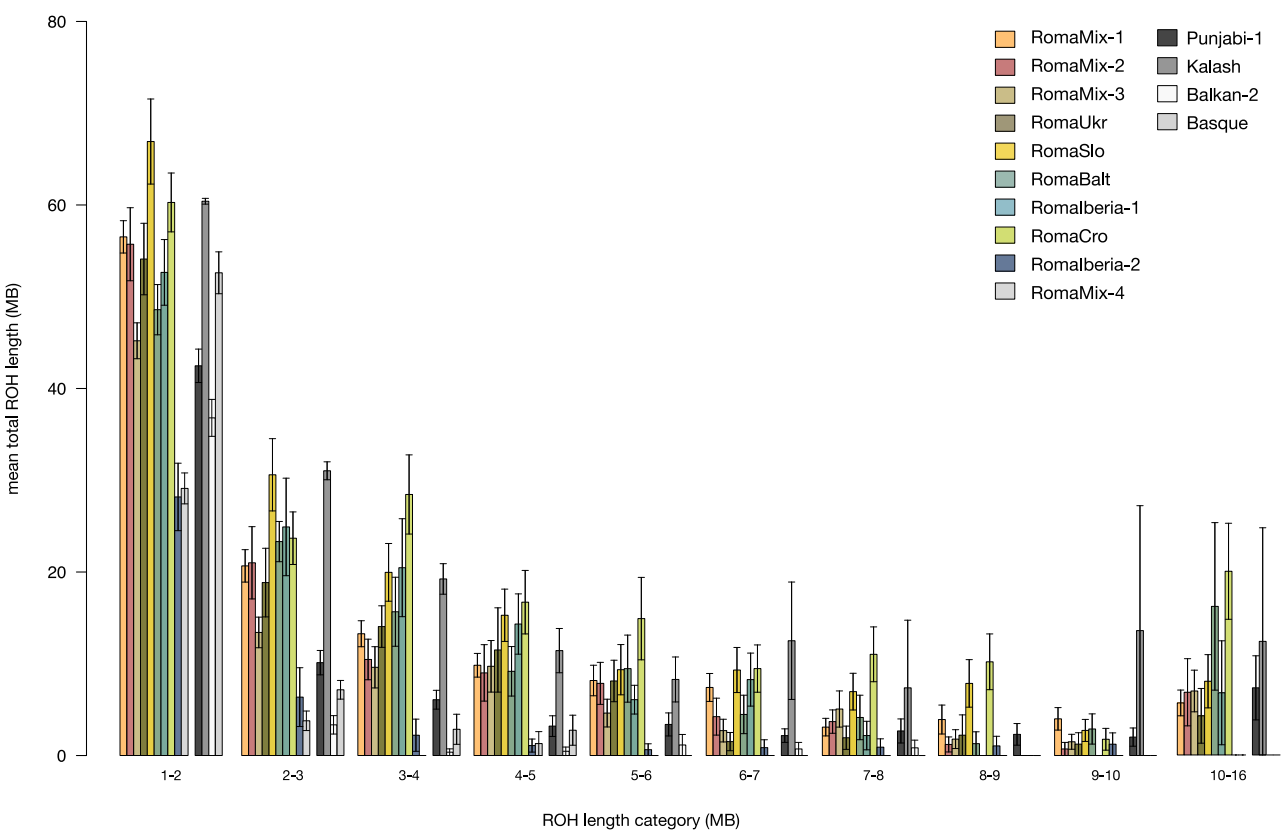

Supplement: S10 Fig — Each vertical bar represents a population group: reference populations (greyish colors) and European Roma clusters. (PDF) [file pgen.1008417.s014.pdf]

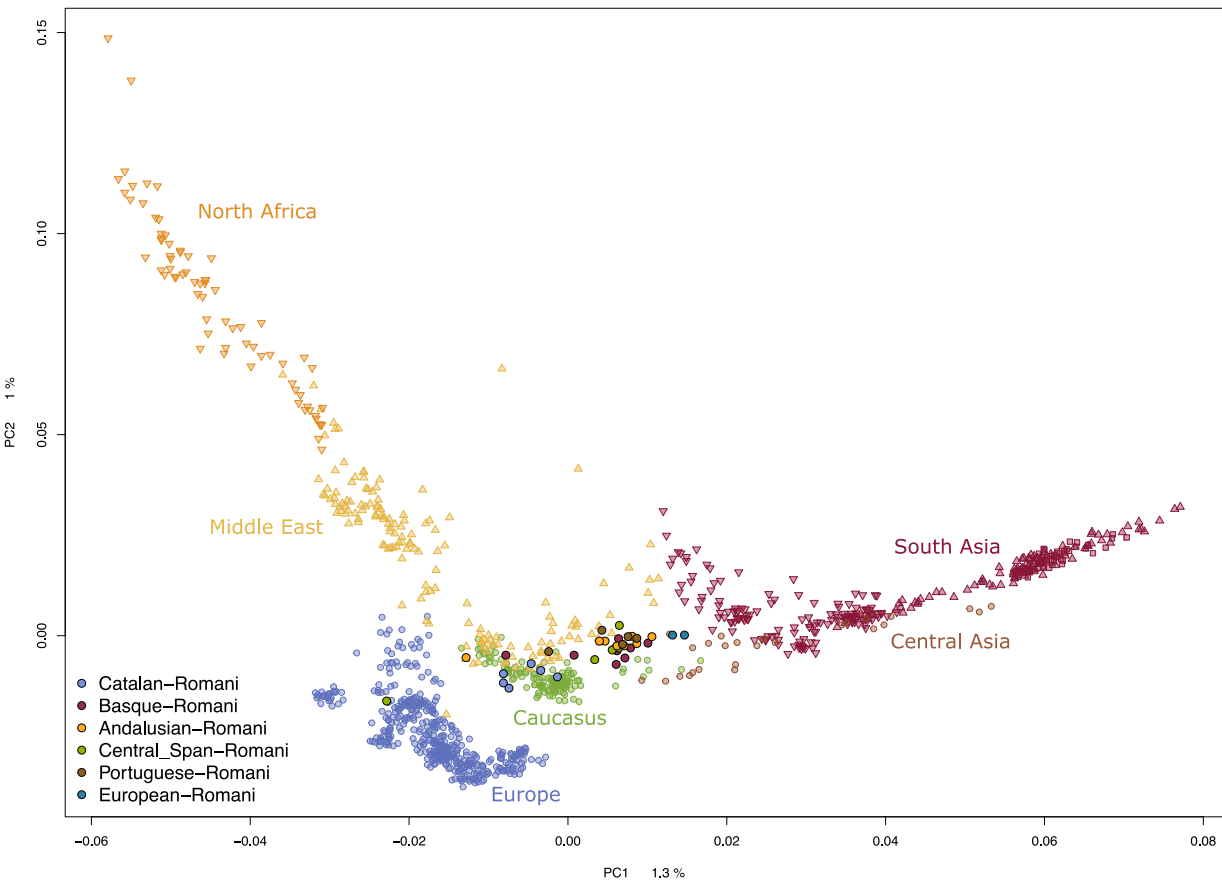

Supplement: S11 Fig — (PDF) [file pgen.1008417.s015.pdf]

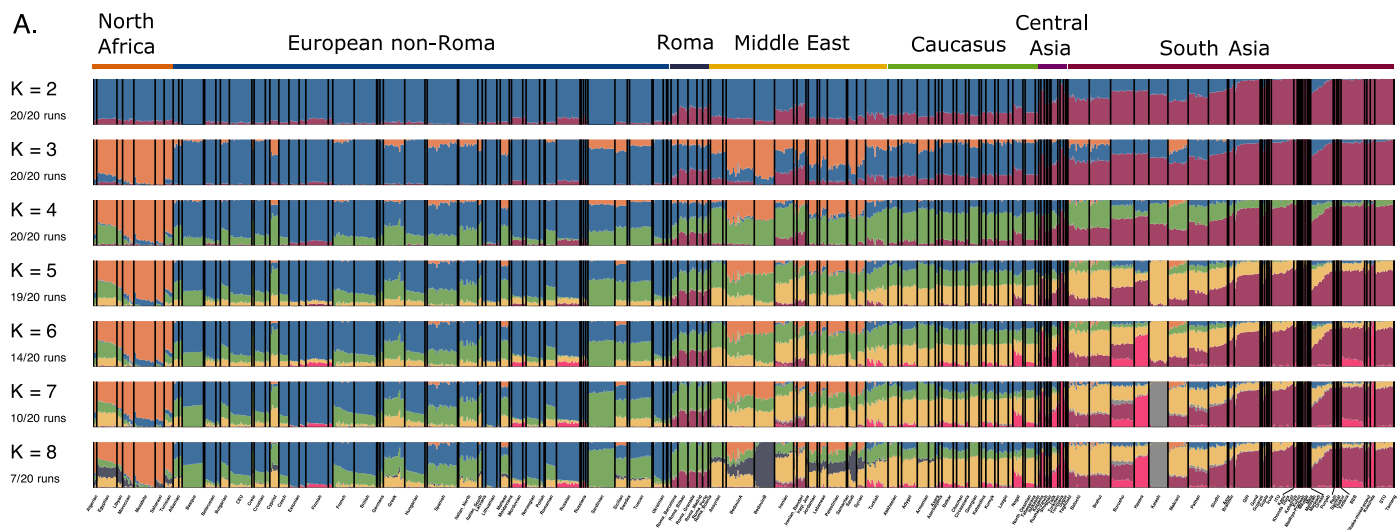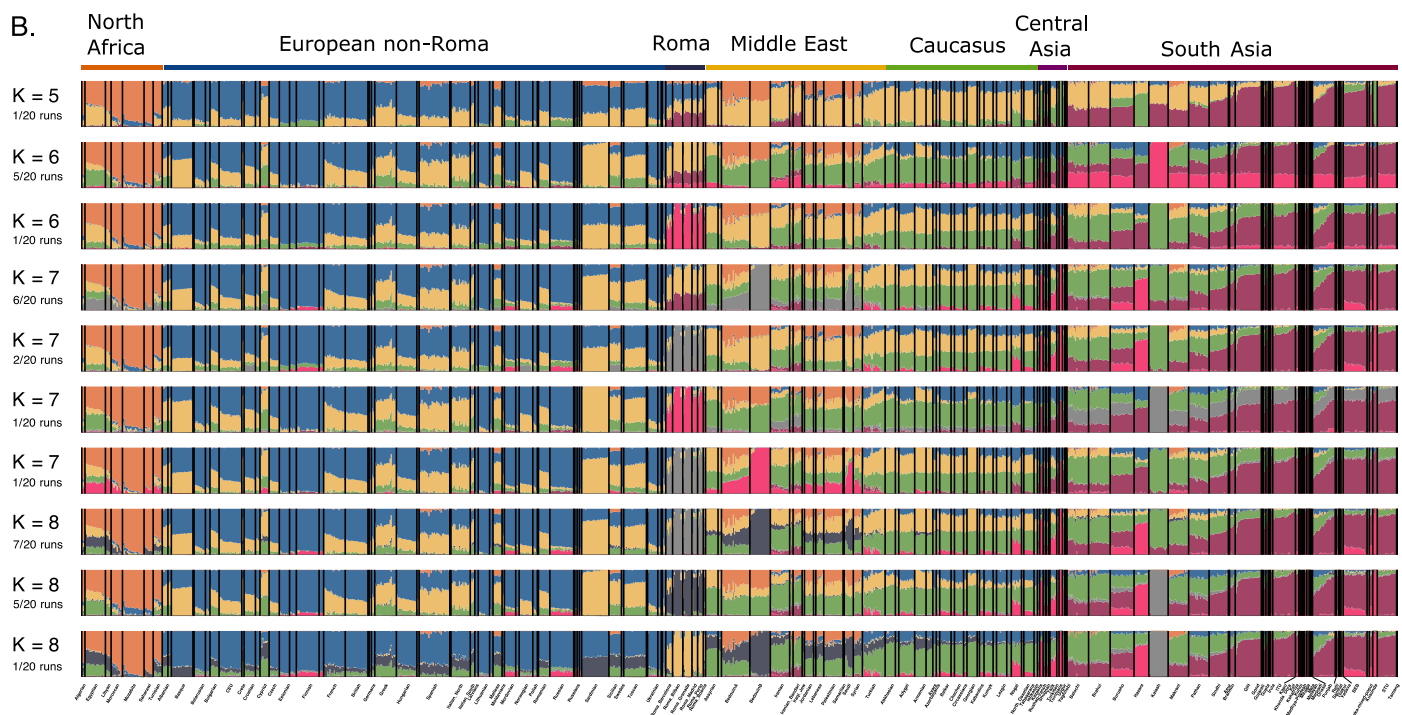

Supplement: S12 Fig — Each vertical line represents one individual and each color represents the proportion of each ancestral component. Major modes are shown in A and minor modes in B. (PDF) [file pgen.1008417.s016.pdf]

ME - Africa

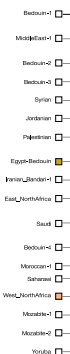

Europe

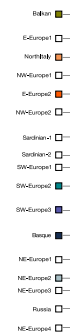

Iberian Roma

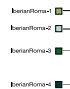

ME - Caucasus

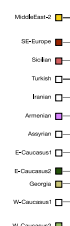

Central and South Asia

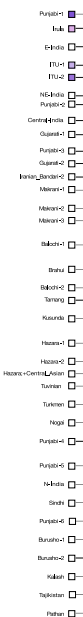

Supplement: S13 Fig — Colored boxes include Iberian Roma clusters and those non-Roma clusters identified as contributing sources to the Roma genomes in the GLOBETROTTER results. (PDF) [file pgen.1008417.s017.pdf]

A.

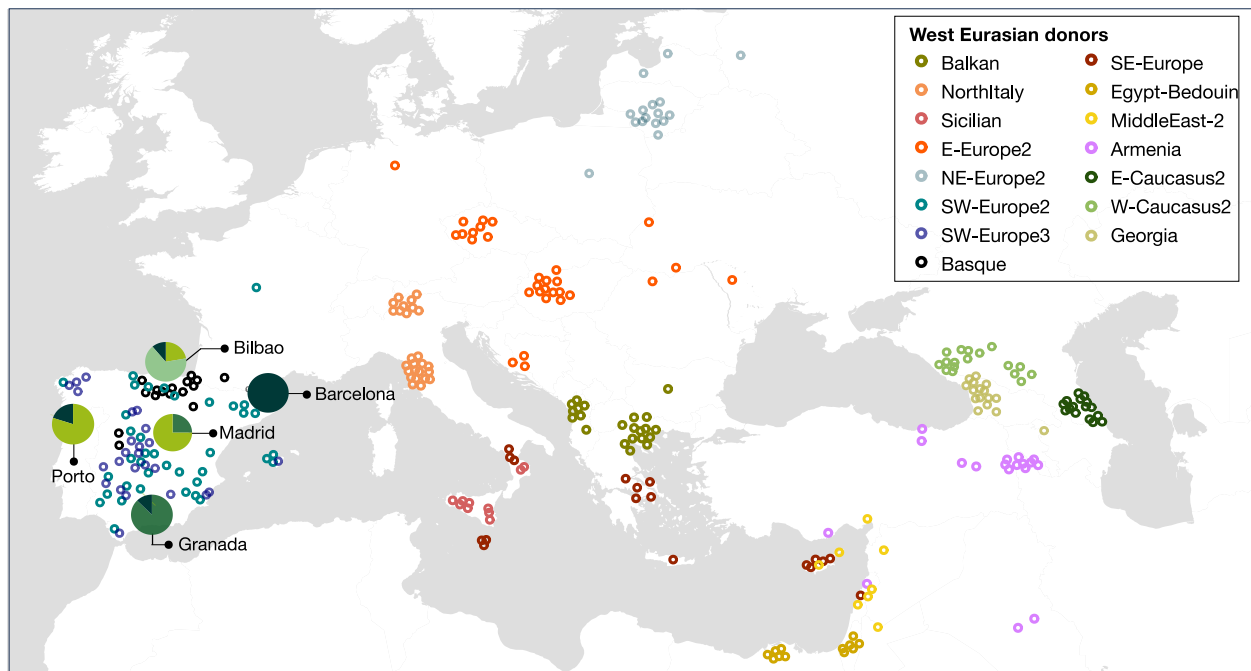

B.

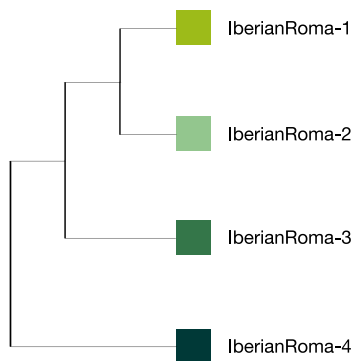

C.

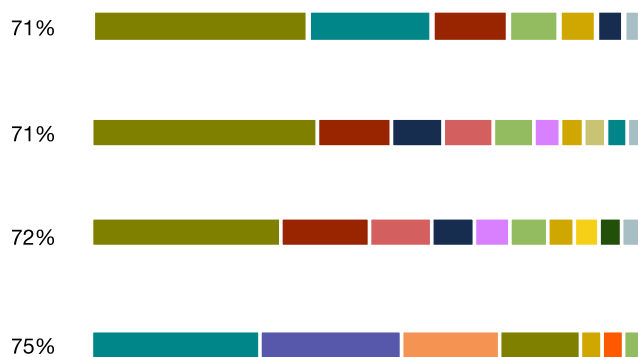

Supplement: S14 Fig — (A) Iberian Roma sampled groups (pie charts are colored according the clusters in B) and their West Eurasian donors in the GLOBETROTTER analysis. (B) Iberian Roma fineSTRUCTURE dendrogam showing the four Iberian Roma clusters. (C) Major source of the admixture event inferred by GLOBETROTTER: for each Roma cluster, the proportion (in percentage) of the major source and a horizontal bar with the proportions of each donor populations (colored as in A), that contribute a minimum of 0.2 to the major source, are shown. (PDF) [file pgen.1008417.s018.pdf]

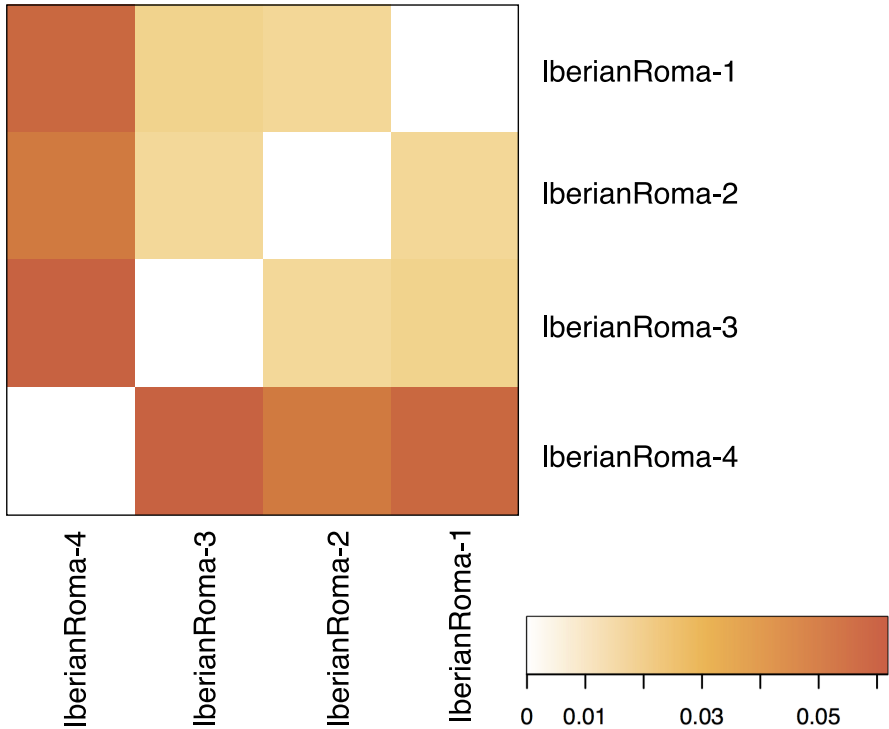

Supplement: S15 Fig — (PDF) [file pgen.1008417.s019.pdf]

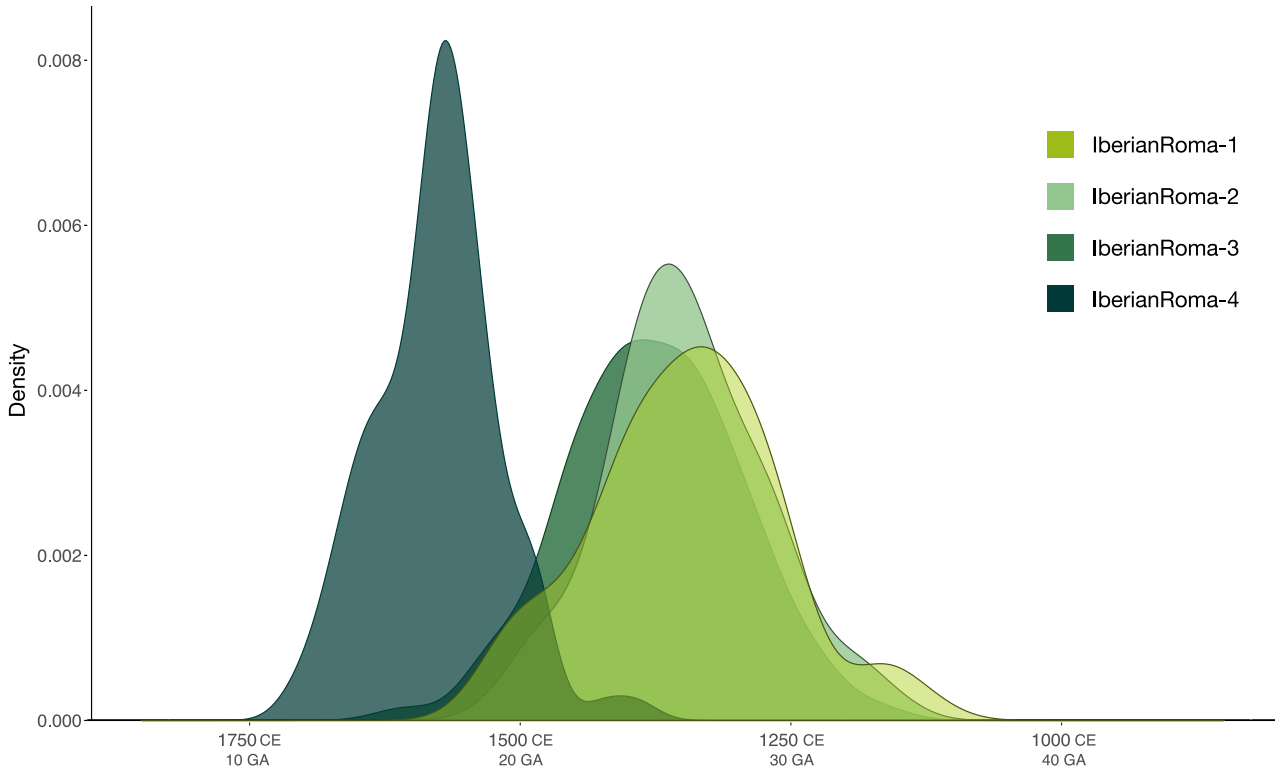

Supplement: S16 Fig — Admixture dates (x-axis) are shown in years CE (assuming a generation time of 25 years) and in generations agp (GA). (PDF) [file pgen.1008417.s020.pdf]

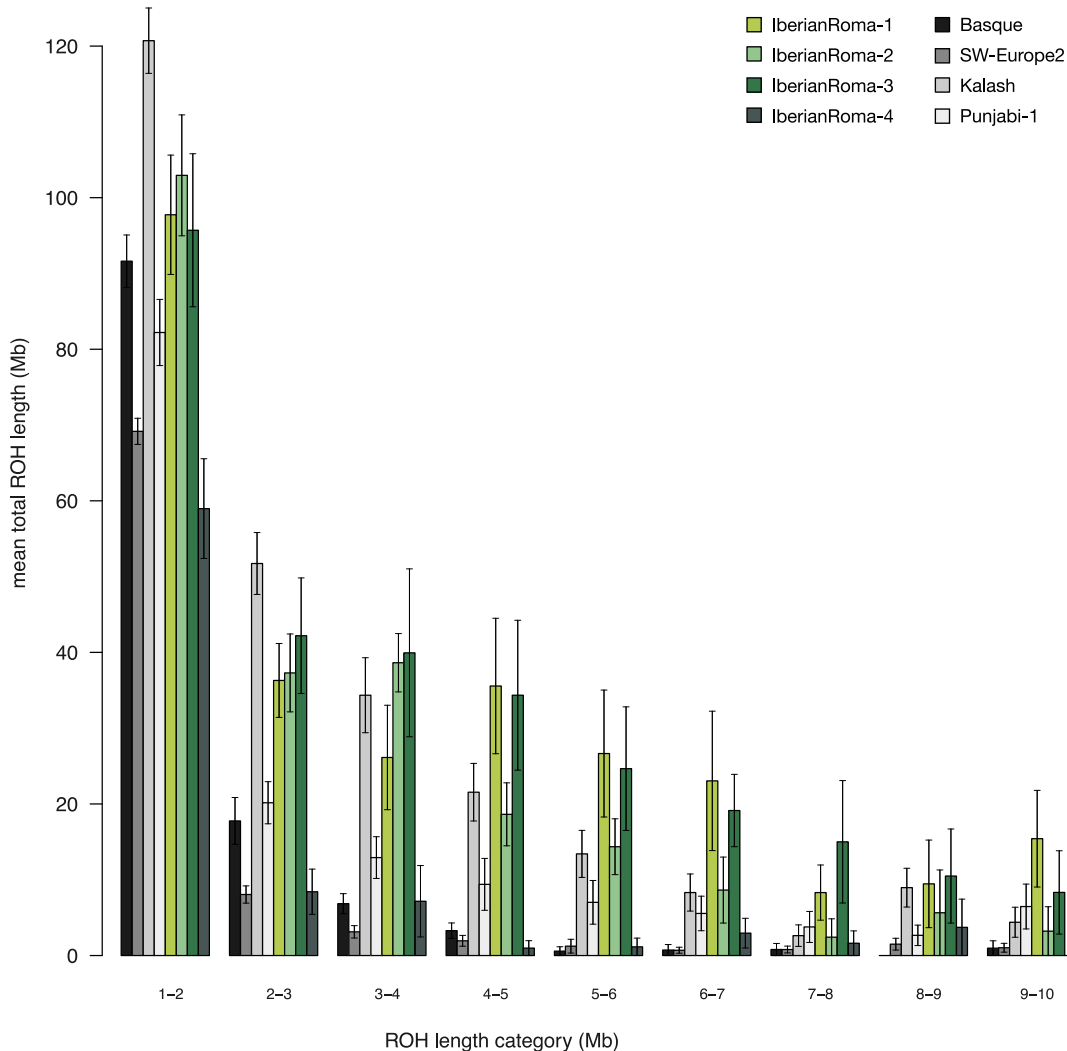

Supplement: S17 Fig — Each vertical bar represents a population group: reference populations (greyish colors) and Iberian Roma clusters (greenish colors). (PDF) [file pgen.1008417.s021.pdf]

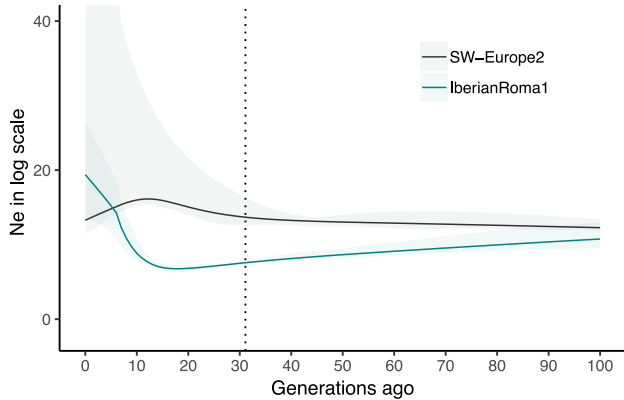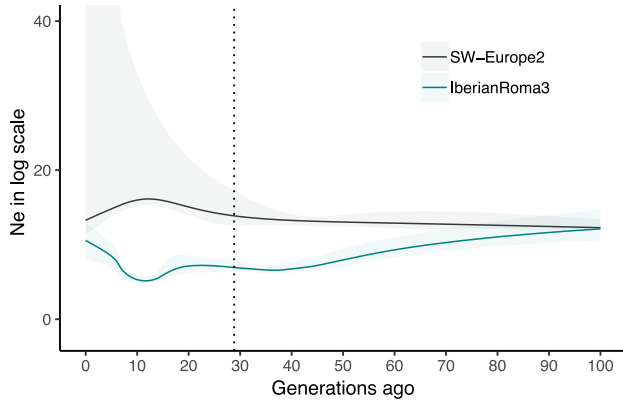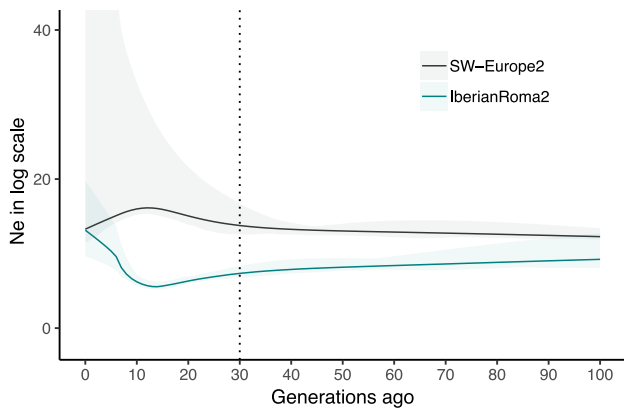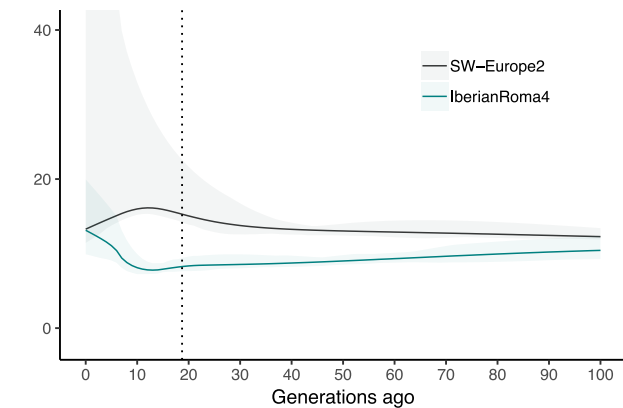

Supplement: S18 Fig — X-axis corresponds to number of generations ago. The vertical dotted lines represent the start of the admixture in each group (lowerCI of the admixture date inferred with GLOBETROTTER). (PDF) [file pgen.1008417.s022.pdf]

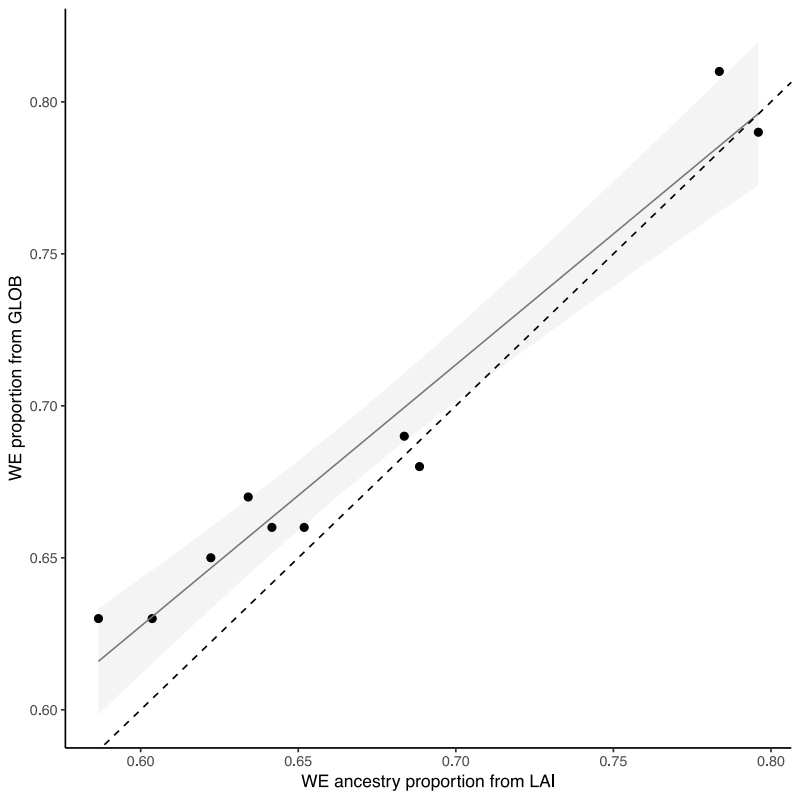

Supplement: S19 Fig — Each dot represents a Roma cluster (Dataset 1). The dashed line represents the line of equality (x = y). (PDF) [file pgen.1008417.s023.pdf]
