# Supplementary material for: European Roma groups show complex West Eurasian admixture footprints and a common South Asian genetic origin
Source: PLoS Genet. 2019 Sep 23;15(9):e1008417. doi: 10.1371/journal.pgen.1008417 (PMC6779411; doi:10.1371/journal.pgen.1008417)
Supplement: S4 Fig — (PDF) [file pgen.1008417.s008.pdf]

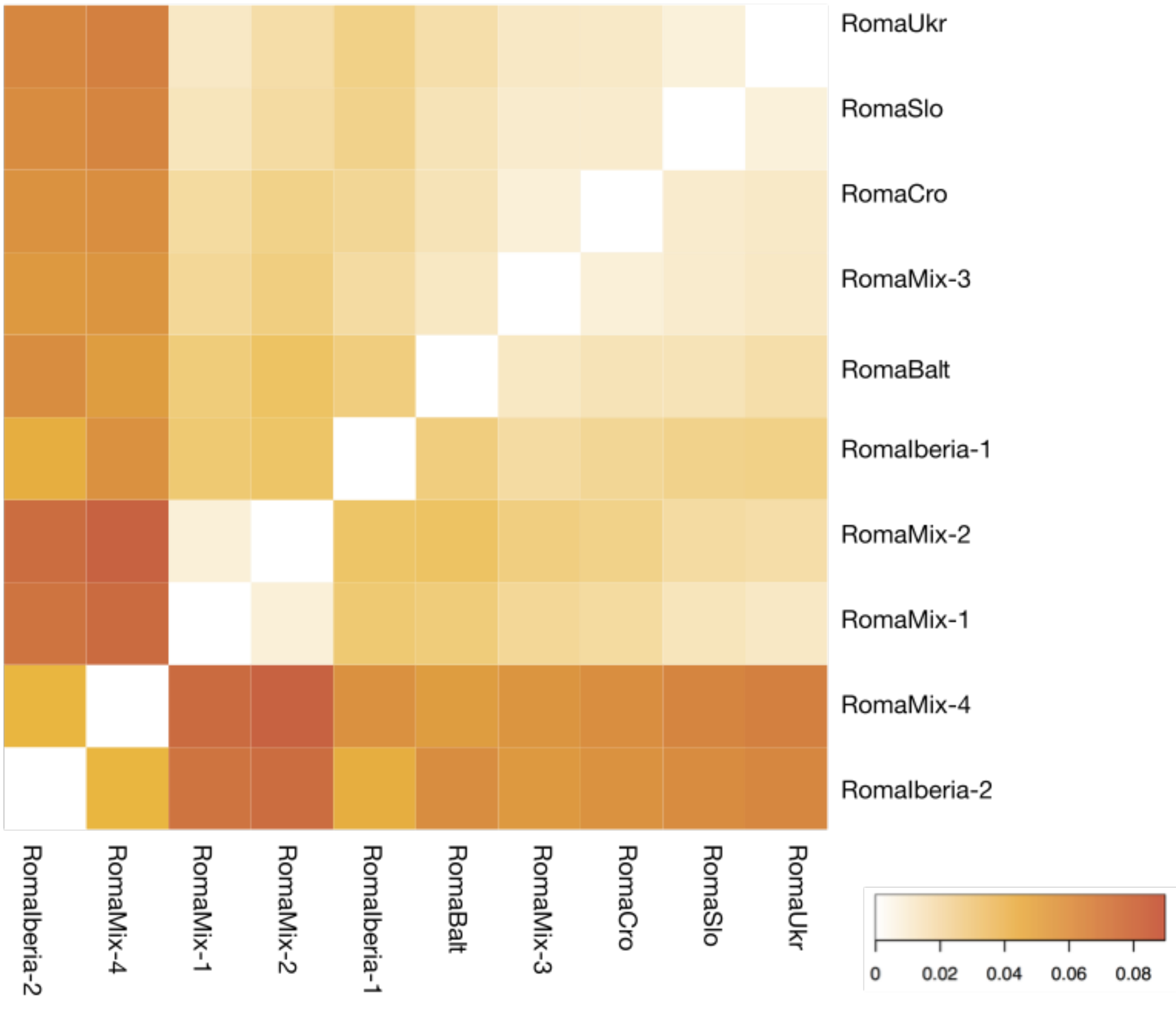
RomaUkr
RomaSlo
RomaCro
RomaMix-3
RomaBalt
RomaIberia-1
RomaMix-2
RomaMix-1
RomaMix-4
RomaIberia-2
RomaIberia-2
RomaMix-4
RomaMix-1
RomaMix-2
RomaIberia-1
RomaBalt
RomaMix-3
RomaCro
RomaSlo
RomaUkr
0
0.02
0.04
0.06
0.08
